# The Intrathecal Analgesia in Robotic Surgery (IARS) Study: Real-World Associations with Intraoperative Opioid Exposure, Fluid Administration, and Postoperative Pain-Free Recovery

**DOI:** 10.3390/clinpract16090167

**Published:** 2026-09-09

**Authors:** Antonio Romanelli, Antonella Langone, Angela Rosaria Caccavale, Alberto D’Amicantonio, Mariafidelia Ferrara, Renato Gammaldi

**Affiliations:** Department of Anesthesia and Intensive Care, A.O.U. “San Giovanni di Dio e Ruggi d’Aragona”, 84131 Salerno, Italy

**Keywords:** spinal anesthesia, levobupivacaine, robotic surgical procedures, analgesics, opioid, remifentanil, enhanced recovery after surgery, pain, postoperative, fluid therapy

## Abstract

**Background/Objectives:** Subarachnoid analgesia (SA) is increasingly used as an adjunct to general anesthesia in robotic ERAS pathways, but the contribution of the intrathecal local anesthetic, as distinct from that of the intrathecal opioids, has not been isolated. **Methods:** This study comprised a single-center retrospective cohort of adults undergoing elective robotic surgery (May 2024–July 2025). The exposure was the intrathecal regimen, serving as mutually exclusive categories. The primary outcome was intraoperative opioid exposure (model-derived area under the concentration–time curve per hour, AUC/h); secondary outcomes were standardized intraoperative fluid administration and a stringent pain-free 48 h course (NRS = 0 throughout). Multivariable Gamma and logistic models were supplemented by provider-adjusted mixed-effects analyses, E-values, and a model-free robustness analysis. **Results:** Of 207 patients, 83.1% received SA; all were given intrathecal morphine and 62.3% levobupivacaine. Compared with no SA, morphine alone was not associated with AUC/h (+14.0%; 95% CI, −0.9 to +31.1), whereas all levobupivacaine-containing regimens were: −36.4% (IM + L5), −24.3% (IM + L5 + S3), and −44.8% (IM + L10 + S3); overall *p* < 0.001. Algebraically equivalent component reparameterization attributed this to levobupivacaine dose (−44.3% at 5 mg; −59.3% at 10 mg). Remifentanil was used in 98.7%, 88.6%, and 10.0% of patients receiving 0, 5, and 10 mg, respectively. Fluid administration was lower with IM + L5 (−24.9%) and IM + L10 + S3 (−31.0%) after adjustment, but was not with levobupivacaine-graded unadjustment. SA was associated with a pain-free course (adjusted OR, 3.33; 95% CI, 1.06–11.79), an estimate not robust to minimal unmeasured confounding. **Conclusions:** Levobupivacaine-containing regimens were consistently associated with lower intraoperative opioid exposure, but regimen and opioid strategy were chosen jointly by the same clinician; therefore, these associations between strategies are specific to the delivery context of this study. The fluid and pain-free findings are hypothesis-generating. Trials should vary the local anesthetic dose independently of intrathecal opioids.

## 1. Introduction

Robotic surgery is increasingly integrated into Enhanced Recovery After Surgery (ERAS) pathways, but prolonged operative times, carbon dioxide pneumoperitoneum, steep Trendelenburg positioning, and limited intraoperative access after robotic docking may contribute to substantial nociceptive and physiological stress [1,2,3,4]. Inadequate management of this nociceptive burden may therefore increase intraoperative opioid requirements and compromise ERAS adherence [2,5,6].

Within multimodal opioid-sparing ERAS strategies, neuraxial techniques, including subarachnoid analgesia (SA), are widely used adjuncts in selected major surgical settings. However, their role in robotic surgery remains debated because of procedure-specific logistics and the potential hemodynamic effects of spinal blockade during pneumoperitoneum and positioning [1,2,3,4,5,7,8,9,10].

Current evidence on SA in robotic surgery is limited and heterogeneous. Randomized studies, mainly in robotic-assisted radical prostatectomy, reported lower opioid consumption and improved postoperative pain control with intrathecal bupivacaine-based regimens [11,12,13,14]. A systematic review confirmed the potential analgesic benefit of intrathecal morphine while highlighting substantial heterogeneity and limited sample sizes [15]. However, available studies largely focus on postoperative outcomes, and little data quantify intraoperative opioid exposure as a pharmacokinetically informed metric rather than as cumulative postoperative consumption across the heterogeneous range of robotic procedures encountered in routine practice.

Levobupivacaine, the S(-)-enantiomer of bupivacaine, offers a comparable sensory motor block with a more favorable cardiovascular toxicity profile [16], yet it has not been specifically characterized as an intrathecal adjunct in robotic surgery. It therefore remains unclear whether intrathecal levobupivacaine-containing regimens translate into measurable intraoperative opioid sparing and whether any such association is dose-dependent.

We therefore conducted a single-center retrospective cohort study, the Intrathecal Analgesia in Robotic Surgery (IARS) study, of patients undergoing heterogeneous robotic procedures within an ERAS-oriented pathway. The primary objective was to evaluate the association between the intrathecal analgesic regimen, analyzed as mutually exclusive treatment categories, and intraoperative opioid exposure, quantified as a model-derived area under the concentration–time curve per hour (AUC/h). Because clinically administered regimens vary jointly in several components, complementary component-based parameterization was also fitted to isolate the contribution of each intrathecal drug.

Secondary objectives were to assess the association between the intrathecal regimen and standardized intraoperative fluid administration, examined as an exploratory, clinician-mediated surrogate for intraoperative hemodynamic management, and to conduct an exploratory analysis of factors associated with a completely pain-free postoperative course during the first 48 h. The cohort reflects routine real-world practice within an established ERAS pathway, and the analysis is hypothesis-generating.

## 2. Materials and Methods

### 2.1. Study Design and Patient Selection

We conducted a single-center retrospective cohort study (A.O.U. “San Giovanni di Dio e Ruggi d’Aragona”, Salerno, Italy) of all consecutive adult patients (≥18 years) who underwent elective robotic surgery between 2 May 2024 and 31 July 2025. Patients with substance use disorder, chronic pain treatment, or conversion to open surgery were excluded, as were cases in which remifentanil was administered but the cumulative dose could not be reliably reconstructed from the anesthetic records. No formal sample size calculation was performed; the cohort included all eligible patients during the study period, and the study is therefore exploratory.

### 2.2. Perioperative Management

Standard perioperative management was conducted according to an institutional ERAS-oriented protocol (Supplementary File). For patients providing specific consent, single-shot SA was performed before general anesthesia (GA) under sterile conditions using a midline approach with a 27-gauge Whitacre needle, with interspace selection depending on the surgical site. All SA patients received intrathecal morphine (minimum 100 mcg); levobupivacaine 0.33% (5.0 or 10.0 mg) and/or sufentanil (3.0 mcg) were added at the anesthesiologist’s discretion. Reasons for not performing SA included patient refusal, contraindications, or the anesthesiologist’s judgment.

GA was induced with midazolam, propofol, fentanyl or sufentanil, and rocuronium, followed by lung-protective ventilation. GA was maintained with sevoflurane or, in selected cases, propofol target-controlled infusion (TCI; Schnider) titrated to BIS 40–60; remifentanil was administered via TCI (Minto) and titrated to hemodynamic responses. Magnesium sulfate (MgSO_4_) was optionally administered as part of the opioid-sparing strategy. Before emergence, multimodal analgesia (paracetamol, ketorolac when not contraindicated, and tramadol) and trocar-site infiltration with 0.5% ropivacaine were administered; ondansetron was given, and neuromuscular blockade was reversed with sugammadex. Postoperatively, all patients received scheduled paracetamol with additional opioid analgesia and rescue therapy delivered according to predefined Numeric Rating Scale (NRS)-based thresholds. Pain at rest was assessed by trained nursing staff using an 11-point NRS at predefined time points from awakening to 48 hours post operation. Detailed protocols are reported in the Supplementary Materials.

### 2.3. Data Collection

Baseline variables included demographics, body mass index (BMI), smoking status, comorbidities (including the Charlson Comorbidity Index [CCI]), ASA Physical Status, and baseline medication burden. Surgical variables included procedure type, surgical site category, and surgical and anesthesia duration. The neuraxial strategy was recorded as the presence of SA and the administered intrathecal drugs; intraoperative adjuncts (MgSO_4_, clonidine, norepinephrine, metoprolol) were also recorded. The requirement for continuous vasopressor infusion and the administration of intraoperative metoprolol were recorded as indicators of major intraoperative hemodynamic instability.

Intraoperative fluid input, urine output, and net balance were normalized to body weight and anesthesia duration. Blood loss was standardized by weight and surgical duration.

Intraoperative opioid exposure was quantified using two complementary metrics. Both incorporated the fentanyl-equivalent induction bolus and the cumulative intraoperative remifentanil dose, and both were standardized to actual body weight and anesthesia duration. The first was the total intraoperative opioid administration rate (ng/kg/min), calculated as the sum of the induction opioid bolus (fentanyl or sufentanil, converted to fentanyl equivalents) and the cumulative dose of remifentanil administered during maintenance, divided by actual body weight and anesthesia duration. The second was a model-derived area under the concentration–time curve per hour (AUC/h, ng·h/mL), in which the same administration data were first converted into estimated concentration–time profiles using first-order pharmacokinetic decay and then integrated [17,18]. Anesthesia duration, rather than surgical duration, was selected for standardization to quantify total opioid exposure throughout the entire anesthetic period. Opioid administration begins during anesthetic induction and may continue until emergence; therefore, anesthesia duration more appropriately represents the time interval over which systemic opioid exposure occurs than surgical duration alone. The two metrics therefore share the same drug set, the same potency conversions, and the same denominator, and differ only in whether drug disposition is modeled; AUC/h served as the primary outcome and the administration rate as a directly observed, model-free alternative. Potency conversions, pharmacokinetic assumptions, and computational details are reported in the Supplementary Materials. Separately, the fentanyl-equivalent dose administered at induction (µg/kg) was recorded as a baseline anesthetic characteristic and retained as a covariate.

The NRS assessed postoperative pain, and perioperative opioid consumption was summarized as morphine milligram equivalents (MME) [19].

### 2.4. Outcomes

The primary outcome was the derived-model area under the concentration–time curve per hour (AUC/h), as described in Section 2.3.

The first secondary outcome was standardized intraoperative fluid administration, calculated as the total volume of intravenous fluids administered from anesthetic induction to the end of anesthesia, including crystalloids, colloids, and blood products, divided by actual body weight and anesthesia duration, and expressed as mL/kg/h.

The second secondary outcome was a completely pain-free postoperative course during the first 48 postoperative hours, defined as an NRS pain score of 0 at rest at all prespecified assessments from emergence to anesthesia to 48 h after surgery (for details, see Supplementary Materials).

Given the retrospective observational design and the non-random allocation of intrathecal analgesic regimens, all analyses should be considered exploratory. The distinction between primary and secondary outcomes reflects the prespecified analytical hierarchy established before statistical modeling and was intended to prioritize interpretation rather than to support confirmatory hypothesis testing.

### 2.5. Statistical Analysis

Continuous variables are reported as the mean ± standard deviation (SD) or median and first-third quartiles (q_1_–q_3_), depending on the data distribution assessed with the Shapiro–Wilk test; categorical variables are presented as counts and percentages.

As an initial descriptive analysis, patients were stratified according to the intrathecal analgesic regimen received. Continuous variables were compared using one-way analysis of variance (ANOVA) or the Kruskal–Wallis test, as appropriate, while categorical variables were compared using the χ^2^ test or Fisher’s exact test when observed cell counts were <5. These comparisons are descriptive and were not adjusted for multiplicity.

Associations between candidate predictors and both AUC/h and standardized intraoperative fluid administration were evaluated using univariate and multivariable generalized linear models with a Gamma distribution and a log link, selected because both outcomes were continuous, strictly positive, and right-skewed; the log link allowed coefficients to be interpreted as proportional changes. The same covariate set was applied to both continuous outcomes and was selected a priori on clinical grounds, before any outcome model was fitted, based on established or plausible determinants of intraoperative opioid requirement and fluid administration. No data-driven selection was performed, and no covariate was added or removed on the basis of univariate results.

The primary analyses evaluated mutually exclusive intrathecal analgesic regimens as the exposure of interest. To better characterize the contribution of individual neuraxial drugs, complementary multivariable models replacing the regimen variable with the individual intrathecal components (morphine, levobupivacaine dose, and sufentanil) were also fitted. Exponentiated regression coefficients were reported as percentage changes with corresponding 95% confidence intervals (95% CIs). Because AUC/h is a model-derived measure, a prespecified robustness analysis was performed using the standardized cumulative intraoperative opioid administration rate (ng/kg/min) as an alternative model-free outcome. Detailed model specifications, diagnostics, and sensitivity analyses are reported in the Supplementary Materials.

To facilitate clinical interpretation of the multivariable models, adjusted estimated marginal means (EMMs) for each intrathecal analgesic regimen were estimated from the fitted Gamma regression models on the response scale. Pairwise comparisons between regimens were subsequently performed using the EMM framework, with *p*-values adjusted for multiple testing according to the Benjamini–Hochberg false discovery rate (FDR) procedure.

Factors associated with a pain-free postoperative course (NRS = 0 during the first 48 postoperative hours) were evaluated using univariate and multivariable logistic regression analyses. Effect estimates are presented as odds ratios (ORs) with 95% confidence intervals (95% CIs). Complete-case analyses were performed for postoperative NRS data, and comparisons between complete and incomplete datasets are reported in the Supplementary Materials. Given the limited number of pain-free events relative to the number of candidate predictors, the multivariate logistic model was considered exploratory, and its estimates were treated as hypothesis-generating.

For all models, multicollinearity among predictors was assessed using generalized variance inflation factors (VIFs; values < 5 considered acceptable by convention), and model assumptions were assessed using simulated residual diagnostics (DHARMa). To account for clustering by anesthesiologist, all primary Gamma and logistic regression models were additionally refitted as mixed-effects models including the attending anesthesiologist as a random intercept.

As a sensitivity analysis, the potential impact of unmeasured confounding on the primary associations was quantified using E-values. For the Gamma regression models, which used a log link, exponentiated regression coefficients were interpreted as multiplicative mean ratios and used to calculate E-values. For the binary outcome, E-values were calculated from adjusted risk ratios estimated using a modified Poisson regression model with a log link and robust standard errors. For effect estimates below 1, the reciprocal of the estimate was used, as originally proposed by VanderWeele and Ding [20]. The E-value represents the minimum strength of association that an unmeasured confounder would need to have with both the exposure and the outcome, conditional on the measured covariates, to fully explain away the observed association. E-values were calculated for both the point estimate and the confidence limit closest to the null.

In light of the findings from the primary analyses, additional post hoc analyses were performed to further investigate and support the clinical interpretation of the observed associations.

All tests were two-sided with α = 0.05, and *p*-values < 0.05 were considered significant. Statistical analyses were conducted using R version 4.5.2 (R Foundation for Statistical Computing). The results are reported in tables and plots.

## 3. Results

### 3.1. Cohort Characteristics

Of the 259 robotic-assisted procedures performed between 2 May 2024 and 31 July 2025, 15 cases were excluded (3 for substance use disorders, 2 for chronic pain treatments, 10 for conversions to open surgery). A further 37 cases were excluded because the remifentanil maintenance dose could not be retrieved despite its administration, yielding a final cohort of 207 patients, seen by five consultant anesthesiologists.

Baseline characteristics and perioperative management are summarized in Table 1.

Briefly, the cohort had a median age of 66.0 years (q_1_–q_3_ 55.0–71.0), was predominantly male (62.3%), and classified as ASA II (65.7%); surgical sites were heterogeneous (pelvic 47.3%, retroperitoneal 18.3%, upper abdominal 17.9%), and Trendelenburg positioning was used in 57.5% of cases. Median surgical and anesthesia times were 210.0 (q_1_–q_3_ 162.5–250.0) and 240.0 (q_1_–q_3_ 195.0–280.0) minutes, respectively. SA was performed in 172 patients (83.1%); all received intrathecal morphine, with levobupivacaine in 129 (62.3%; 5.0 mg in 38.2% and 10.0 mg in 24.1%) and sufentanil in 86 (41.5%). Propofol TCI was used in 6.8% of cases, MgSO_4_ in 53.6% (median 11.8 mg/kg), and clonidine in 11.6% of cases. Remifentanil infusion was used in 73.4% of cases, with a median rate of 31.1 ng/kg/min (q_1_–q_3_ 15.5–54.9). Median AUC/h was 25.87 ng·h/mL (q_1_–q_3_ 19.73–36.04). Median standardized fluid administration was 5.37 mL/kg/h (q_1_–q_3_ 3.96–6.59), urine output was 0.74 mL/kg/h (q_1_–q_3_ 0.49–1.10), and standardized blood loss was 0.58 mL/kg/h (q_1_–q_3_ 0.42–0.79). No patient required a continuous norepinephrine infusion, and no intraoperative metoprolol was administered. No clinically documented adverse events related to SA were identified during the postoperative hospital stay. Specifically, no cases of respiratory depression requiring intervention, clinically significant motor block, neurological complications, pruritus requiring treatment, or urinary retention attributable to the intrathecal technique were recorded.

### 3.2. Baseline and Perioperative Characteristics According to Intrathecal Analgesia Regimen

Regarding SA regimen, 35 patients (16.9%) did not receive SA (No-SA group), 43 patients (20.8%) received intrathecal morphine alone (IM group, minimum dose 100 mcg, maximum dose 250 mcg, mean dose 1.68 mcg/kg, SD 0.45 µg/kg), 43 (20.8%) received intrathecal morphine (fixed dose 100 µg) combined with levobupivacaine 5 mg (IM + L5 group), 36 (17.4%) received intrathecal morphine (fixed dose 100 µg) combined with levobupivacaine 5 mg and sufentanil 3 µg (IM + L5 + S3 group), and 50 (24.1%) received intrathecal morphine (fixed dose 100 µg) combined with levobupivacaine 10 mg and sufentanil 3 µg (IM + L10 + S3 group). Baseline demographic, clinical, and perioperative characteristics by intrathecal analgesic regimen are reported in Table 2.

Significant between-regimen differences were observed in several clinical, procedural, and anesthetic variables. The distribution of solid malignancy differed across groups (χ^2^ test *p* = 0.008), with localized tumors more frequent in the IM + L10 + S3 group (80.0%) and less frequent in the No-SA group (45.7%). CCI comorbidity also differed modestly among regimens (Kruskal–Wallis test *p* = 0.046), although the median was 2 points in all groups. The operated organ differed significantly across regimens (χ^2^ test *p* = 0.021): gallbladder procedures were more frequent in the No-SA and IM + L5 + S3 groups, whereas prostate surgery was more frequent in the IM + L5 and IM + L10 + S3 groups. Trendelenburg positioning also varied significantly (χ^2^ test *p* = 0.004), ranging from 31.4% in the No-SA group to 74.4% in the IM + L5 group.

Surgical and anesthesia durations differed significantly across regimens (both Kruskal–Wallis tests, *p* < 0.001). Patients in the No-SA group had the shortest median surgical and anesthesia durations (165 and 193 min, respectively), whereas the IM group had the longest (225 and 250 min).

The induction opioid was strongly associated with the selected intrathecal regimen (χ^2^ test *p* < 0.001). Fentanyl was administered to all patients in the IM and IM + L5 groups; sufentanil was administered to all patients in the IM + L5 + S3 and IM + L10 + S3 groups. The fentanyl-equivalent induction dose also differed across regimens (Kruskal–Wallis test *p* = 0.009): the highest median dose was observed in the IM + L5 group and the lowest in the IM and IM + L10 + S3 groups. Substantial differences were also observed in intraoperative co-interventions: MgSO_4_ and clonidine administration (both χ^2^ test *p* < 0.001).

Remifentanil infusion was used in nearly all patients in the No-SA, IM, and IM + L5 groups, but in only 77.8% of the IM + L5 + S3 group and 10.0% of the IM + L10 + S3 group (χ^2^ test *p* < 0.001). Among patients who received intraoperative remifentanil (*n* = 152), the infusion rate differed significantly by intrathecal analgesic regimen (Kruskal–Wallis test, *p* < 0.001). Patients managed without intrathecal levobupivacaine showed the highest remifentanil infusion rates, with median values of 49.8 (q_1_–q_3_ 28.2–64.9) ng/kg/min in the No-SA group and 50.5 (q_1_–q_3_ 30.4–75.9) ng/kg/min in the IM group. In contrast, regimens including intrathecal levobupivacaine were associated with substantially lower infusion rates, with median values of 19.6 (q_1_–q_3_ 13.2–32.9) ng/kg/min for IM + L5, 18.7 (q_1_–q_3_ 11.7–28.9) ng/kg/min for IM + L5 + S3, and 8.6 (q_1_–q_3_ 8.2–10.2) ng/kg/min for IM + L10 + S3. However, the estimate for the IM + L10 + S3 group should be interpreted cautiously, as only five patients in this group required remifentanil infusion.

Model-derived intraoperative opioid exposure differed significantly across regimens (Kruskal–Wallis test, *p* < 0.001). The median AUC/h was highest in the IM group at 38.28 ng·h/mL and lowest in the IM + L10 + S3 group at 16.90 ng·h/mL. Colloid administration also differed across groups (χ^2^ test *p* = 0.002), being most frequent in the IM + L5 + S3 group.

Finally, standardized fluid administration and balance differed significantly among regimens (both Kruskal–Wallis tests, *p* < 0.001). The lowest median fluid administration was observed in the IM + L5 group at 4.00 mL/kg/h, whereas the highest was observed in the No-SA group at 5.99 mL/kg/h. Similarly, median fluid balance was lowest in the IM + L5 group and highest in the No-SA and IM + L5 + S3 groups.

### 3.3. Association Between Intrathecal Analgesic Regimen and Opioid AUC/h

Table 3 reports univariate and multivariable Gamma regression analyses of the association between intrathecal analgesic regimen and opioid AUC/h.

In the adjusted model, increasing age was independently associated with lower AUC/h (−0.5% per year, 95% CI −0.9 to −0.1; *p* = 0.021), whereas current smoking was associated with higher opioid exposure (+17.2%, 95% CI +6.2 to +29.5; overall *p* = 0.002).

The intrathecal analgesic regimen was independently associated with model-derived intraoperative opioid exposure (overall *p* < 0.001). Compared with No-SA, IM was not associated with a significant difference in AUC/h (+14.0%, 95% CI −0.9 to +31.1), whereas regimens containing levobupivacaine were associated with significantly lower opioid exposure. Specifically, IM + L5, IM + L5 + S3, and IM + L10 + S3 were associated with reductions in AUC/h of 36.4% (95% CI 26.6 to 45.0), 24.3% (95% CI 4.5 to 40.6), and 44.8% (95% CI 31.0 to 56.3), respectively, compared with No-SA.

Propofol TCI (+40.2%, 95% CI +19.4 to +65.3; *p* < 0.001) and clonidine administration (+20.0%, 95% CI +4.9 to +37.5; *p* = 0.008) were independently associated with a higher AUC/h. The coefficient for the fentanyl-equivalent induction dose (+47.5%, 95% CI +35.9 to +60.4) is reported in Table 3 for completeness only. Because the induction bolus is itself a component of the AUC/h outcome, this term is structural (part–whole) and is not interpreted as a clinical association here or elsewhere in the manuscript.

In the sensitivity analyses using E-values, compared with No-SA, the regimens showed the following values: the IM regimen, E-value of 1.54 (confidence limit E-value = 1.00); the IM + L5 regimen, E-value of 2.52 (confidence limit E-value = 2.07); the IM + L5 + S3 regimen, E-value of 1.97 (confidence limit E-value = 1.27); the IM + L10 + S3 regimen, E-value of 3.03 (confidence limit E-value = 2.26).

When the individual components of the neuraxial analgesic regimen were examined as separate covariates, intrathecal levobupivacaine was the only component independently associated with AUC/h, demonstrating a clear dose–response relationship (overall *p* < 0.001). Compared with no intrathecal levobupivacaine, doses of 5.0 mg and 10.0 mg were associated with reductions in AUC/h of 44.3% (95% CI 36.2 to 51.3) and 59.3% (95% CI 51.3 to 66.0), respectively. In contrast, neither intrathecal morphine nor intrathecal sufentanil was independently associated with AUC/h (see Supplementary Materials).

Sensitivity analyses using E-values supported the robustness of the observed association between intrathecal levobupivacaine and AUC/h. Compared with no levobupivacaine, administration of 5 mg yielded an E-value of 2.99 (confidence limit E-value = 2.51); administration of 10 mg yielded an E-value of 4.35 (confidence limit E-value = 3.53).

The robustness analysis using the standardized intraoperative opioid administration rate as the outcome and the provider-adjusted mixed-effects analysis both yielded results consistent with those of the primary regimen-based analysis, confirming the direction and magnitude of the observed associations. The complementary drug component analysis also showed a consistent pattern of results (Supplementary Materials).

**Figure 1:**
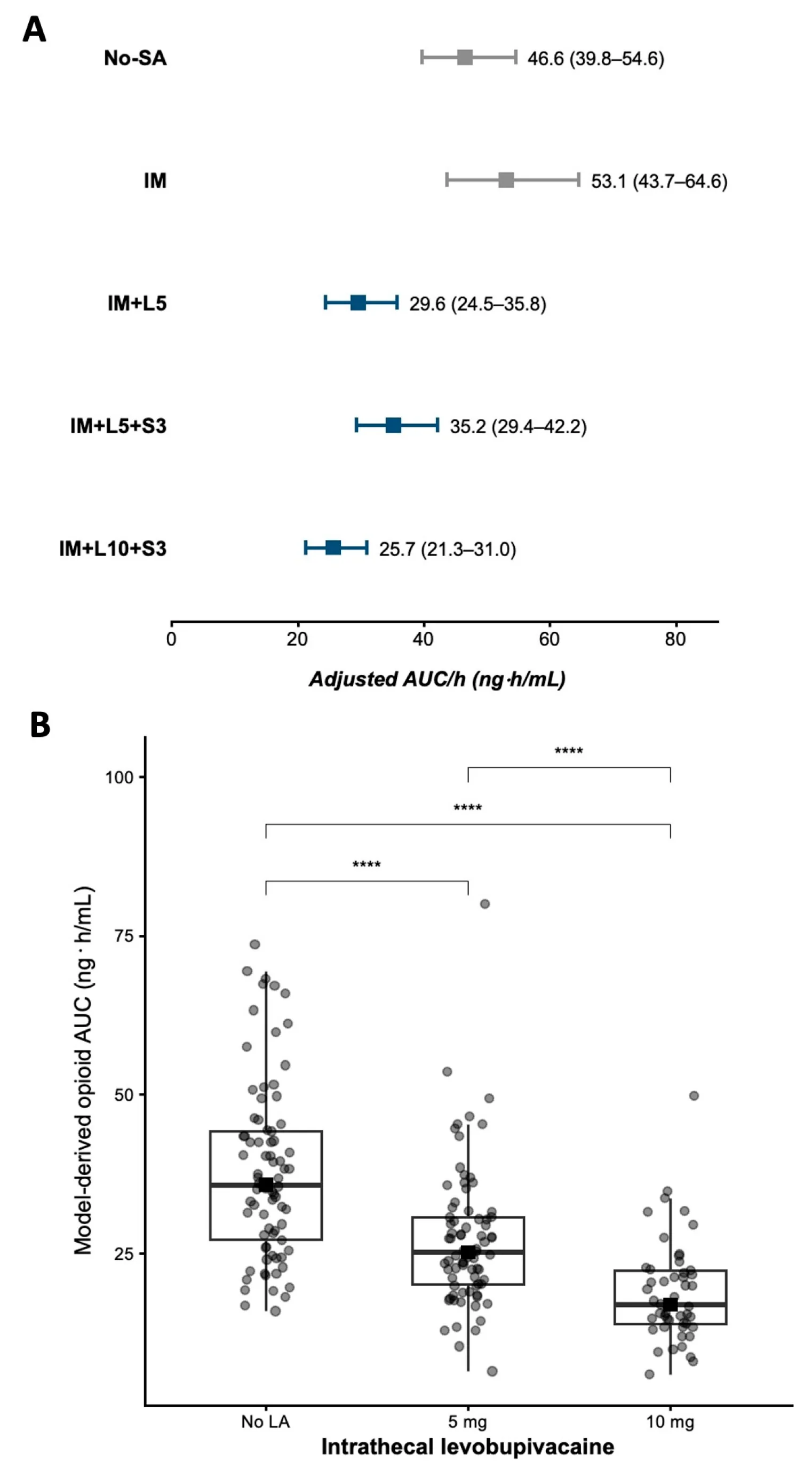
The association between intrathecal analgesic regimen, intrathecal levobupivacaine dose, and model-derived intraoperative opioid exposure. (**A**) Adjusted estimated marginal means of model-derived intraoperative opioid exposure (AUC/h) according to intrathecal analgesic regimen, obtained from the multivariable Gamma regression model. Squares represent adjusted estimated marginal means, and horizontal bars indicate 95% confidence intervals. Gray symbols indicate regimens without intrathecal levobupivacaine, whereas blue symbols indicate regimens containing intrathecal levobupivacaine. (**B**) The unadjusted distribution of model-derived intraoperative opioid AUC/h according to intrathecal levobupivacaine dose (0, 5, or 10 mg). Boxes represent the median and interquartile range (IQR); whiskers extend to 1.5 × IQR, and individual observations are displayed as jittered points. Pairwise comparisons were performed using false discovery rate (FDR) correction. A significant dose-dependent reduction in model-derived intraoperative opioid exposure was observed with increasing intrathecal levobupivacaine dose. ****: *p*-value < 0.001.

FDR-adjusted pairwise comparisons of the EMMs (Figure 1A) showed that AUC/h was significantly lower in all levobupivacaine-containing regimens than in the IM group. Compared with IM, AUC/h was lower with IM + L5 (ratio 0.557; adjusted *p* < 0.001), IM + L5 + S3 (ratio 0.664; adjusted *p* = 0.009), and IM + L10 + S3 (ratio 0.484; adjusted *p* < 0.001). Compared with No-SA, AUC/h was also significantly lower with IM + L5 (ratio 0.636; adjusted *p* < 0.001), IM + L5 + S3 (ratio 0.756; adjusted *p* = 0.034), and IM + L10 + S3 (ratio 0.552; adjusted *p* < 0.001). No significant difference was observed between No-SA and IM (adjusted *p* = 0.089).

Among the levobupivacaine-containing regimens, IM + L10 + S3 was associated with a significantly lower AUC/h than IM + L5 + S3 (ratio 0.729; adjusted *p* < 0.001), whereas no significant differences were observed between IM + L5 and IM + L5 + S3 (adjusted *p* = 0.254) or between IM + L5 and IM + L10 + S3 (*p* = 0.313).

A post hoc unadjusted analysis was performed to further explore the association between intrathecal levobupivacaine dose and model-derived AUC/h. A significant dose–response relationship was observed (Kruskal–Wallis test *p* < 0.001, Figure 1B). The edian AUC/h progressively decreased with increasing intrathecal levobupivacaine dose, from 35.7 ng·h/mL (q_1_–q_3_ 27.2–44.3) in patients who did not receive intrathecal levobupivacaine to 25.1 ng·h/mL (q_1_–q_3_ 20.2–30.6) in those receiving 5 mg, and 16.9 ng·h/mL (q_1_–q_3_ 13.9–22.2) in those receiving 10 mg. Post hoc pairwise comparisons using FDR correction confirmed significant differences between all dose categories (0 vs. 5 mg: adjusted *p* < 0.001; 0 vs. 10 mg: adjusted *p* < 0.001; 5 vs. 10 mg: adjusted *p* < 0.001), supporting a progressive dose-dependent reduction in model-derived intraoperative opioid exposure with increasing intrathecal levobupivacaine dose.

The proportion of patients receiving intraoperative remifentanil differed significantly by intrathecal levobupivacaine dose (χ^2^ test, *p* < 0.001). Remifentanil was administered in 98.7% of patients who did not receive levobupivacaine, 88.6% of those receiving 5 mg, and only 10.0% of those receiving 10 mg. Pairwise comparisons confirmed significant differences among all groups: No LA versus 5 mg (adjusted *p* = 0.023), No LA versus 10 mg (adjusted *p* < 0.001), and 5 mg versus 10 mg (adjusted *p* < 0.001). This indicates a marked dose-dependent reduction in intraoperative remifentanil use.

### 3.4. Association Between Intrathecal Analgesic Regimen and Standardized Intraoperative Fluid Administration

Table 3 reports univariate and multivariable Gamma regression analyses of the association between intrathecal analgesic regimen and standardized intraoperative fluid administration.

In the adjusted model, BMI was independently associated with lower standardized intraoperative fluid administration, with a 2.9% reduction for each 1 kg/m^2^ increase (95% CI −4.0 to −1.8; *p* < 0.001). Similarly, a higher CCI comorbidity score was associated with lower fluid administration (−7.0% per point, 95% CI −11.1 to −2.6; *p* = 0.002).

Intrathecal regimen remained independently associated with standardized fluid administration (overall *p* < 0.001). Compared with the No-SA group, IM + L5 was associated with a 24.9% reduction (95% CI −36.3 to −11.5), whereas IM + L10 + S3 was associated with a 31.0% reduction (95% CI −47.1 to −11.1). No significant differences were observed for IM alone or IM + L5 + S3 after adjustment.

Greater intraoperative standardized blood loss was independently associated with higher fluid administration (+18.1%, 95% CI +9.2 to +28.9; *p* < 0.001).

Sensitivity analyses using E-values indicated moderate robustness of the associations between intrathecal analgesic regimens and standardized intraoperative fluid administration. Compared with No-SA, the IM regimen yielded an E-value of 1.41 (confidence limit E = 1.00); the IM + L5 regimen yielded an E-value of 1.99 (confidence limit E-value = 1.51); the IM + L5 + S3 regimen yielded an E-value of 1.71 (confidence limit E-value = 1.00). The largest E-value was observed for the IM + L10 + S3 regimen (2.26, confidence limit E-value = 1.50).

When the individual components of the neuraxial analgesic regimen were examined as separate covariates, intrathecal levobupivacaine was the only component independently associated with intraoperative standardized fluid administration, demonstrating a clear dose–response relationship (overall *p* < 0.001). Compared with no levobupivacaine, 5.0 mg and 10.0 mg were associated with reductions of −17.9% (95% CI −29.6 to −4.1) and −31.5% (95% CI −44.1 to −16.0), respectively. The sensitivity analyses using E-values reported the following results: compared with no levobupivacaine, administration of 5 mg yielded an E-value of 1.73 (confidence limit E-value = 1.26); administration of 10 mg yielded an E-value of 2.28 (confidence limit E-value = 1.67).

The provider-adjusted mixed-effects analysis yielded results consistent with the primary multivariable model. Model diagnostics and details are reported in the Supplementary Materials.

**Figure 2:**
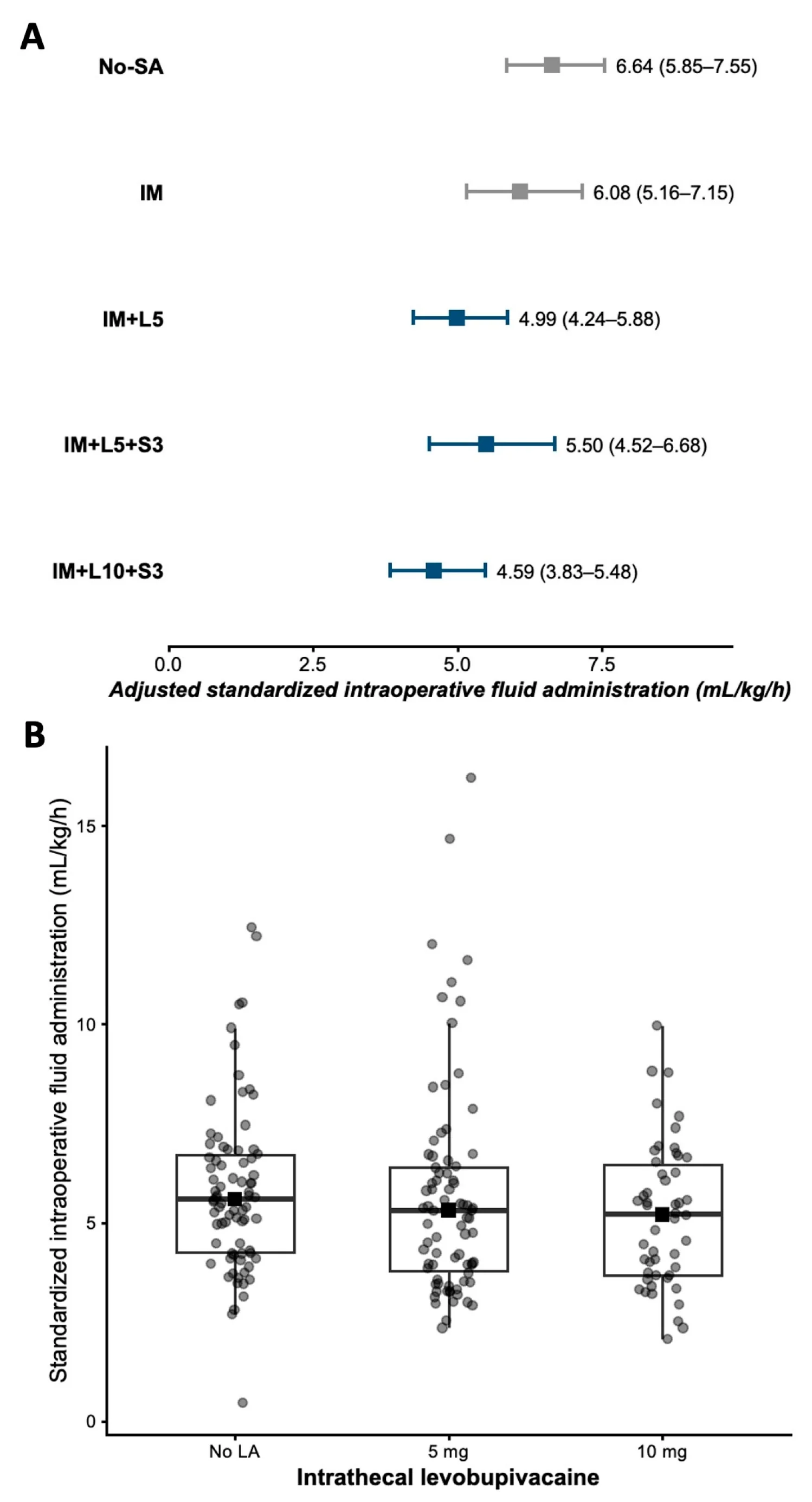
The association between intrathecal analgesic regimen, intrathecal levobupivacaine dose, and standardized intraoperative fluid administration. (**A**) Adjusted estimated marginal means (EMMs) of standardized intraoperative fluid administration according to intrathecal analgesic regimen, obtained from the multivariable Gamma regression model. Squares represent adjusted EMMs, and horizontal bars indicate 95% confidence intervals. Gray symbols indicate regimens without intrathecal levobupivacaine, whereas blue symbols indicate regimens containing intrathecal levobupivacaine. (**B**) The unadjusted distribution of standardized intraoperative fluid administration according to intrathecal levobupivacaine dose (0, 5, or 10 mg). Boxes represent the median and interquartile range (IQR), whiskers extend to 1.5 × IQR, and individual observations are displayed as jittered points. No significant differences were observed in the unadjusted comparison among levobupivacaine dose groups (Kruskal–Wallis test, *p* = 0.167). However, multivariable adjustment demonstrated an independent dose-dependent association between intrathecal levobupivacaine and lower standardized intraoperative fluid administration.

FDR-adjusted pairwise comparisons of the EMMs (Figure 2A) confirmed the results of the multivariable model. Compared with the No-SA group, standardized intraoperative fluid administration was significantly lower in the IM + L5 (ratio 0.751; adjusted *p* = 0.007) and IM + L10 + S3 (ratio 0.690; adjusted *p* = 0.030) groups, whereas no significant difference was observed for IM + L5 + S3 (adjusted *p* = 0.290). Similarly, IM + L5 was associated with lower standardized fluid administration compared to IM (ratio 0.821; adjusted *p* = 0.038). No significant differences were observed between IM and IM + L10 + S3 (adjusted *p* = 0.149), between IM and IM + L5 + S3 (adjusted *p* = 0.594), or between IM + L5 and IM + L10 + S3 (adjusted *p* = 0.594). Among the levobupivacaine-containing regimens, only IM + L10 + S3 showed lower standardized fluid administration compared to IM + L5 + S3 (ratio 0.834; adjusted *p* = 0.044).

A post hoc unadjusted analysis was performed to further explore the association between intrathecal levobupivacaine dose and standardized intraoperative fluid administration. In contrast to model-derived intraoperative opioid exposure, standardized intraoperative fluid administration did not differ significantly across intrathecal levobupivacaine dose categories (Kruskal–Wallis *p* = 0.167, Figure 2B). Median standardized fluid administration was 5.59 mL/kg/h (q_1_–q_3_ 4.25–6.71) in patients who did not receive intrathecal levobupivacaine, 5.31 mL/kg/h (q_1_–q_3_ 3.79–6.40) in those receiving 5 mg, and 5.21 mL/kg/h (q_1_–q_3_ 3.69–6.47) in those receiving 10 mg.

### 3.5. Postoperative Pain

Overall, 64 patients (30.9%) required additional postoperative opioid analgesia. Of these, 51 (24.6%) received continuous analgesia via an elastomeric pump, whereas 13 (6.3%) required rescue tramadol because of inadequately controlled pain. The median time-weighted standardized postoperative MME consumption was 4.25 mcg/kg/h (q_1_–q_3_ 2.83–6.07).

Postoperative opioid requirements differed markedly across intrathecal analgesic regimens (χ^2^ test *p* < 0.001). Postoperative opioids were administered to 68.6% of patients in the No-SA group and 53.5% in the IM group, compared with 20.9% in IM + L5, 19.4% in IM + L5 + S3, and only 2.0% in IM + L10 + S3.

Complete postoperative NRS data were available for 159 patients (76.8%). Comparisons between patients with complete and incomplete NRS data are reported in the Supplementary Materials.

The median NRS values were as follows: 0.0 (q_1_–q_3_ 0.0–0.0, min–max 0.0–8.0) at awakening; 0.0 (q_1_–q_3_ 0.0–0.0, min–max 0.0–10.0) after 30 min; 0.0 (q_1_–q_3_ 0.0–0.0, min–max 0.0–8.0) after 1 h; 0.0 (q_1_–q_3_ 0.0–0.0, min–max 0.0–10.0) after 3 h; 0.0 (q_1_–q_3_ 0.0–0.0, min–max 0.0–6.0) after 6 h; 0.0 (q_1_–q_3_ 0.0–0.0, min–max 0.0–7.0) after 12 h; 0.0 (q_1_–q_3_ 0.0–0.0, min–max 0.0–9.0) after 24 h; 0.0 (q_1_–q_3_ 0.0–0.0, min–max 0.0–10.0) after 36 h; 0.0 (q_1_–q_3_ 0.0–0.0, min–max 0.0–6.0) after 48 h.

**Figure 3:**
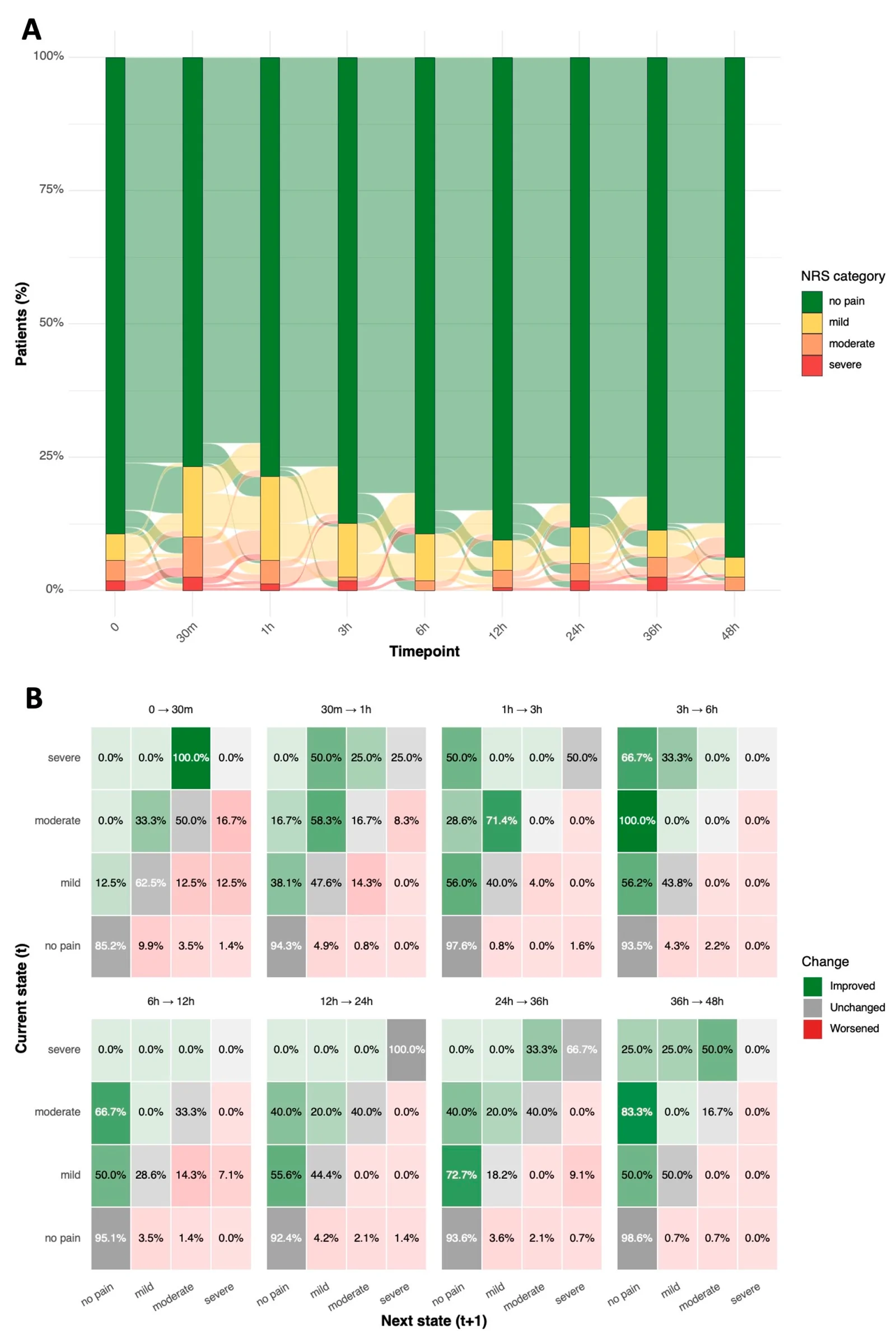
The distribution of postoperative pain categories and observed transitions between consecutive assessments during the first 48 h. Panel (**A**) shows the temporal distribution and transitions of postoperative pain categories using an alluvial plot based on rest NRS assessments. Pain categories were defined as no pain (NRS = 0), mild pain (NRS 1–3), moderate pain (NRS 4–6), and severe pain (NRS ≥ 7). The width of the flows reflects the proportion of patients transitioning between pain categories between consecutive postoperative time points. Panel (**B**) shows observed transition probability matrices describing the probability of transitioning from each pain category at time t to the subsequent category at time t + 1 across consecutive postoperative intervals. Green cells indicate improvement toward lower pain categories, gray cells indicate unchanged pain status, and red cells indicate transitions toward higher pain categories. Overall, most patients remained within the no-pain category throughout follow-up, while transitions toward severe pain were uncommon and generally unstable over time. NRS, Numeric Rating Scale.

The distribution of postoperative pain categories at the nine scheduled assessments and the observed transitions are shown in Figure 3. In the alluvial representation (Panel A), the no-pain category accounted for the majority of the cohort at every time point, and most patients who were pain-free at one assessment remained so at the next; therfore, the dominant flow across the 48 h period was the persistence of the no-pain state. The observed transition matrices (Panel B) show the same pattern in probabilistic terms: the no-pain category had the highest probability of persistence between consecutive assessments, whereas the moderate (NRS 4–6) and severe (NRS ≥ 7) categories were unstable, being more frequently followed by a transition toward a lower category than by persistence. Episodes of moderate-to-severe pain were therefore uncommon and predominantly transient rather than sustained.

**Figure 4:**
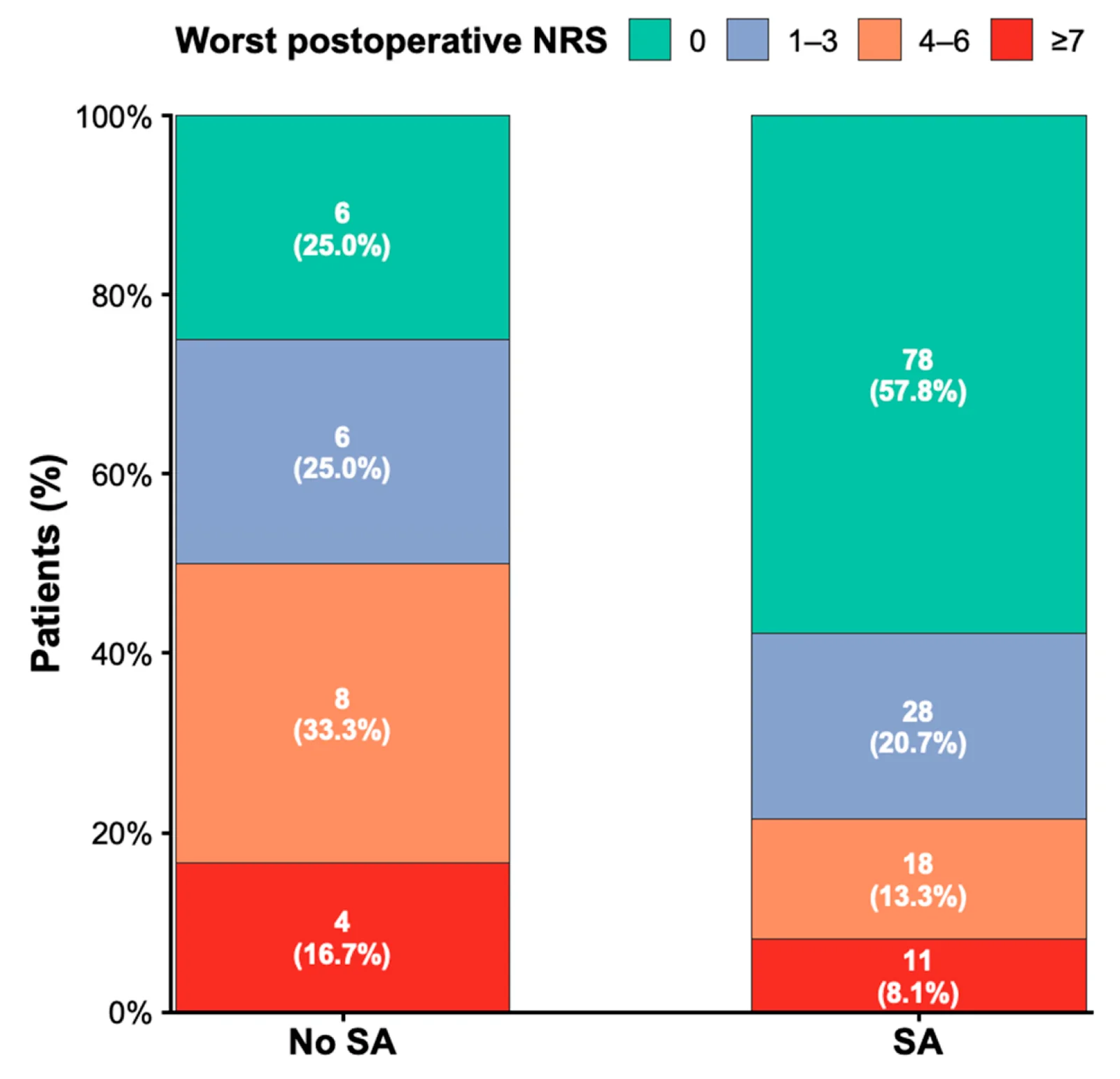
The distribution of the worst postoperative pain intensity during the first 48 postoperative hours according to the use of subarachnoid analgesia (SA). Stacked bars show the proportion of patients in each category of the worst Numeric Rating Scale (NRS) score recorded during the 48 h postoperative follow-up (0, 1–3, 4–6, and ≥7). Values within the bars indicate the number of patients and the corresponding percentage within each group. Patients receiving SA showed a clear shift toward lower postoperative pain severity, with a higher proportion remaining pain-free (worst NRS = 0) and a lower proportion experiencing moderate (NRS 4–6) or severe pain (NRS ≥ 7) compared with patients who did not receive SA (χ^2^ test, *p* = 0.012).

Among the 159 patients with complete postoperative NRS assessments, 24 (15.1%) did not receive SA, whereas 135 (84.9%) underwent SA. Overall, 84 (52.8%) remained pain-free throughout the 48 h follow-up (worst NRS = 0). The remaining patients experienced at least one episode of postoperative pain, with the worst NRS of 1–3 in 34 patients (21.4%), 4–6 in 26 (16.4%), and ≥7 in 15 (9.4%). The distribution of the worst postoperative pain score differed significantly by SA use (χ^2^ test, *p* = 0.012; Figure 4). Among patients who did not receive SA, only 25.0% remained pain-free throughout the 48 h follow-up, compared with 57.8% of those receiving spinal anesthesia. Conversely, moderate pain (worst NRS 4–6) occurred in 33.3% versus 13.3% of patients, while severe pain (worst NRS ≥7) occurred in 16.7% versus 8.1% of patients. Overall, the distribution of postoperative pain severity shifted toward lower pain categories in patients receiving SA.

Table 4 reports univariate and multivariable logistic regression analyses of the association between SA and a pain-free postoperative course.

In the multivariable logistic regression analysis, SA remained independently associated with a pain-free postoperative course during the first 48 h. Patients receiving SA had approximately three-fold higher odds of remaining pain-free than those managed without SA (adjusted OR 3.33, 95% CI 1.06–11.79; *p* = 0.040). The magnitude of the association was attenuated compared with the unadjusted estimate (OR 4.11, 95% CI 1.61–11.92; *p* = 0.003), although the confidence interval remained wide, indicating limited precision.

CCI comorbidity was also independently associated with pain-free status (adjusted OR 1.52 per point, 95% CI 1.03–2.32; *p* = 0.034). We note that postoperative elastomeric pump use was not associated with pain-free status (adjusted OR 1.26, 95% CI 0.49–3.40; *p* = 0.640).

The provider-adjusted pain-free model showed a pattern of associations consistent with the primary analysis; for details, see the Supplementary Materials.

Sensitivity analysis using E-values showed that for the association between neuraxial analgesia and pain-free recovery at 48 h, the adjusted risk ratio was 1.97 (95% CI 0.98–3.95), corresponding to an E-value of 3.35 (confidence limit E-value = 1.00).

## 4. Discussion

In this single-center cohort of heterogeneous robotic procedures managed within an ERAS-oriented pathway, three findings emerged, differing substantially in the strength of the evidence supporting them. Intrathecal regimens containing levobupivacaine were associated with markedly lower intraoperative opioid exposure than no SA, whereas IM alone was not; this association was dose-graded, was present without adjustment, and remained robust to moderate unmeasured confounding. Two of the three levobupivacaine-containing regimens were also associated with lower standardized intraoperative fluid administration, but only after multivariable adjustment; we regard this as provisional. SA, when analyzed as a global neuraxial strategy, was associated with a higher likelihood of a completely pain-free course over 48 h; this estimate was imprecise, not robust to minimal unmeasured confounding, and exploratory.

The regimen-based and component-based models converge on a single interpretation: intrathecal LA, and not morphine or sufentanil, accounts for the reduction in intraoperative opioid exposure. Regimens without levobupivacaine (No-SA and IM) did not differ from one another, whereas every levobupivacaine-containing regimen had significantly lower exposure than IM. In the component model, levobupivacaine 5.0 and 10.0 mg were associated with a progressively lower AUC/h, whereas neither IM nor sufentanil was independently associated with the outcome. This gradient was also observed in unadjusted and provider-adjusted analyses. The ordering is evident in the component and dose category analyses rather than across the five mutually exclusive regimens, since IM + L5 + S3 showed a smaller adjusted reduction than IM + L5 despite an identical levobupivacaine dose.

Intraoperative opioid exposure was driven largely by whether a remifentanil infusion was started at all: remifentanil was administered to 98.7% of patients receiving no intrathecal levobupivacaine, to 88.6% of those receiving 5 mg, and to only 10.0% of those receiving 10 mg. That decision and the choice of intrathecal regimen were made by the same clinician, frequently as components of a single anesthetic plan formulated before incision. AUC/h therefore reflects, in part, a co-determined intraoperative strategy rather than a titrated physiological response to nociception. Because induction opioid choice was largely determined by the regimen, its coefficient is not independently interpretable.

The dose–response gradient persisted in all analyses, and the E-values suggested moderate robustness to unmeasured confounding. However, provider random-intercept models cannot account for within-provider patient-level selection.

The direction of the association is mechanistically plausible. Pneumoperitoneum and steep Trendelenburg positioning increase sympathetic drive and nociceptive afferent input even under GA, and a sufficiently dense neuraxial local anesthetic block may attenuate this signaling and reduce the need for opioid co-titration [8,16,21]. The null intraoperative effect of IM is consistent with this account on pharmacodynamic rather than kinetic grounds: with a median anesthesia duration of 240 min, IM had ample time to reach peak effect, but spinal opioids modulate dorsal horn transmission without producing the dense afferent blockade and sympatholysis that shape the hemodynamic responses to which remifentanil was titrated. Reducing the remifentanil burden may be desirable in itself, since higher or prolonged exposure has been linked to postoperative pain amplification [22,23,24], although we did not measure an endpoint to test this, and the clinical magnitude of the phenomenon remains debated.

Our results are consistent with randomized trials of SA as an adjunct to GA in robotic surgery. In robotic-assisted radical prostatectomy, intrathecal bupivacaine combined with sufentanil or with morphine reduced intraoperative analgesic requirements [12,13,14], and a comparable opioid-sparing effect was reported with IM plus bupivacaine in robot-assisted hysterectomy [11]. These studies are heterogeneous in regimen, indication, and anesthetic protocol; all used bupivacaine, and none was designed to separate the contribution of LA from that of the intrathecal opioids. The novelty of our analysis lies in the concurrent modeling of mutually exclusive regimens and individual intrathecal components, which localized the opioid-sparing association to the local anesthetic and its dose across different robotic procedures. Among alternative regional adjuncts, fascial plane blocks provide somatic, dermatomally restricted analgesia without sympathetic blockade and have shown only modest effects on intraoperative opioid exposure [25,26,27], whereas SA achieves a denser, segmentally broader block at the cost of obligate sympatholysis; our data do not permit a direct comparison, but they support procedure-specific head-to-head trials with intraoperative opioid exposure and standardized hemodynamic endpoints as co-primary outcomes.

The inverse association between intrathecal levobupivacaine and standardized intraoperative fluid administration runs counter to the classical expectation that LA-induced sympathetic blockade should increase the number of clinician-administered fluid boluses [28].

Contemporary practice increasingly favors vasopressors over fluid loading for spinal-induced hypotension, although the supporting evidence is heterogeneous and derives largely from obstetric anesthesia, where the physiological context differs from that of anesthetized patients in Trendelenburg [29,30]. One plausible explanation for our finding is that improved nociceptive control attenuated sympathetic fluctuations and reduced the perceived need for rescue fluids.

We nevertheless regard this finding as provisional. Standardized fluid administration was not dose-graded across levobupivacaine categories in unadjusted analyses, and the association emerged primarily after multivariable adjustment. Moreover, the corresponding E-values were smaller than those observed for AUC/h, indicating greater sensitivity to model specification and unmeasured confounding. Differences in colloid use may partly explain the anomalous pattern observed in the IM + L5 + S3 group. Moreover, fluid administration is clinician-mediated and reflects anticipated nociceptive burden, surgical characteristics, and fluid-type preference rather than hemodynamic stability as such; no patient required continuous vasopressor infusion, but this cannot substitute for the hypotension duration, vasopressor bolus, and bradycardia metrics that were not available.

The association between SA and a pain-free postoperative course is biologically plausible, given the established role of neuraxial analgesia and intrathecal opioids in postoperative pain control [31,32,33,34,35], but the statistical support in our cohort was limited. The estimate was based on a small No-SA reference group and a relatively parameter-rich model, making it susceptible to sparse-data bias. Moreover, the confidence limit E-value indicated sensitivity to minimal unmeasured confounding.

The endpoint was intentionally stringent, requiring an NRS score of 0 at all nine scheduled assessments over 48 h. Because the median NRS was 0 at every time point, classification depended on isolated pain episodes and was sensitive to assessment intensity, documentation variability, rescue thresholds, and non-random missingness. The model evaluated SA as a global strategy rather than as individual intrathecal components due to the limited number of available events.

Several non-neuraxial covariates merit comment and are hypothesis-generating rather than interpretable effects. Increasing age was associated with lower AUC/h (−0.5% per year), consistent with the well-documented age-related reduction in opioid requirement and, for remifentanil specifically, with age-dependent pharmacokinetics and pharmacodynamics in which both clearance and the concentration producing half-maximal effect decline substantially with advancing age [36]. Current smoking was associated with a higher AUC/h, in line with meta-analytic evidence that smokers require approximately one-third more opioids than non-smokers in the early postoperative period [37] and with the altered nociceptive processing described in chronic tobacco exposure [38]. Propofol TCI and clonidine were each associated with a higher rather than lower AUC/h (+40.2% and +20.0%), a direction opposite to the opioid-sparing effect established for perioperative systemic α_2_ agonists [39]; we read this as confounding by indication [40], witg these agents having been selected in cases anticipated to be more challenging, rather than as a pronociceptive effect. Most counterintuitively, a higher CCI comorbidity score was associated with both lower fluid administration and higher odds of a pain-free course. We propose no physiological mechanism for the latter; selection and documentation are more parsimonious: patients with greater comorbidity burden were plausibly offered less extensive procedures, managed more conservatively, and assessed against different rescue thresholds. This estimate is not evidence that comorbidity protects against postoperative pain.

Any opioid-sparing benefit should be balanced against recovery-related effects of a denser neuraxial block, including residual motor block, urinary retention, and hypotension. No SA-related adverse events were documented in this cohort; however, surveillance was retrospective, and PONV was not systematically recorded. The trade-off between opioid sparing and recovery-related outcomes therefore remains uncertain.

Model diagnostics were reassuring regarding fit: adjusted VIFs remained below conventional thresholds, DHARMa-simulated residual diagnostics did not detect major violations of model assumptions, and provider-adjusted mixed-effects models yielded estimates consistent with the primary analyses.

### Limitations

This study has several limitations. First, its retrospective single-center design limits causal inference and generalizability. Baseline and perioperative characteristics differed across groups, including tumor stage, surgical site, Trendelenburg positioning, procedure and anesthesia duration, induction opioid dose, and use of MgSO_4_ and clonidine. Therefore, the comparisons reflect clinical strategies rather than isolated pharmacological interventions.

Second, 37 of 244 eligible patients (15.2%) were excluded because cumulative remifentanil exposure could not be reconstructed. Because remifentanil contributed to the primary outcome, these patients could not be included, and their baseline characteristics were not retained in a separate dataset; consequently, selection bias related to this exclusion cannot be formally assessed.

Third, AUC/h relies on simplified pharmacokinetic assumptions and should be interpreted as an internally consistent exposure index rather than a direct estimate of plasma opioid concentrations. Both exposure metrics include the induction opioid bolus, which was also entered as a covariate; therefore, its coefficient is partly structural and is not interpreted independently. The model-free sensitivity analysis yielded directionally consistent, although larger, estimates, supporting the robustness of the findings to pharmacokinetic modeling while not addressing uncertainties related to opioid-equivalence assumptions or exposure composition.

Fourth, remifentanil was titrated based on hemodynamic responses rather than dedicated nociceptive monitoring, potentially introducing provider-related variability. Intrathecal morphine dosing also differed between regimens, whereas morphine was modeled as a binary variable; however, it was not independently associated with AUC/h.

Fifth, several covariates, including propofol TCI, MgSO_4_, clonidine, induction opioid dose, and blood loss, may have been influenced by the selected anesthetic strategy and could therefore represent mediators rather than true confounders. Adjustment for these variables may have attenuated the estimated overall effect of the neuraxial strategy.

Sixth, the pain-free analysis was based on complete NRS data for 159 of 207 patients (76.8%), with evidence that missingness was non-random. Moreover, other clinically relevant ERAS outcomes, including mobilization, bowel recovery, length of stay, complications, readmission, and PONV, were not consistently or prospectively documented.

Finally, provider-level adjustment was limited to five attending anesthesiologists. Although the random-intercept model accounts for average differences between providers, it cannot fully address patient-level treatment selection or unmeasured intraoperative factors influencing opioid and fluid titration. Residual confounding by indication, therefore, remains possible.

## 5. Conclusions

Within an ERAS-focused robotic surgical pathway, intrathecal regimens with levobupivacaine were linked to significantly lower intraoperative opioid use compared to No-SA or IM. A dose-dependent pattern persisted in unadjusted analyses and remained stable despite moderate unmeasured confounding. Since the intrathecal regimen and intraoperative opioid strategy were chosen jointly by the same clinician, this association should be viewed as a comparison of anesthetic approaches as administered. Confirming a causal opioid-sparing effect would need a randomized trial controlling for confounding by indication. The link to lower standardized intraoperative fluid administration appeared only after multivariable adjustment and was not dose-graded in unadjusted comparisons. This finding should be seen as provisional. In exploratory analyses, SA as a comprehensive neuraxial strategy was associated with pain-free postoperative recovery, but due to a small reference group and minimal unmeasured confounding, this should be regarded as hypothesis-generating rather than definitive.

These results highlight the potential importance of structured neuraxial opioid-sparing strategies within robotic ERAS pathways. They also demonstrate the challenge of isolating the perioperative effects of individual intrathecal components during routine practice, where regimens tend to vary together rather than independently. Future prospective randomized trials or carefully designed comparative effectiveness studies should evaluate predefined, mutually exclusive neuraxial regimens. These should vary the local anesthetic dose independently of intrathecal opioid components, include direct hemodynamic measurements and vasopressor-equivalent metrics, and systematically record ERAS-related recovery outcomes such as motor block, time to mobilization, urinary retention, and PONV.

## Figures and Tables

**Table 1:** Baseline characteristics, perioperative management, and intraoperative outcomes of the study cohort (*n* = 207).

Variable		Results	Min–Max
Age (years)		66.0 (55.0–71.0)	18.0–87.0
Sex, male (%)		129 (62.3)	-
BMI (kg/m^2^)		26.3 (24.2–29.9)	17.6–43.4
Comorbidities			
Solid Tumor	No (%)	62 (29.9)	-
	Localized (%)	142 (68.6)	-
	Metastatic (%)	3 (1.4)	-
Hypertension (%)		130 (62.8)	-
Smoking	No (%)	111 (53.6)	-
	Former (%)	48 (23.2)	-
	Current (%)	48 (23.2)	-
Obesity (%)		52 (25.1)	-
COPD (%)		39 (18.8)	-
Diabetes	No (%)	181 (87.4)	-
	Uncomplicated (%)	24 (11.6)	-
	End-organ injury (%)	2 (1.0)	-
Myocardial infarction (%)		13 (6.3)	-
Atrial fibrillation (%)		9 (4.3)	-
Autoimmune disease (%)		8 (3.9)	-
CKD (%)		4 (1.9)	-
PVD (%)		4 (1.9)	-
CVA (%)		4 (1.9)	-
Mild cognitive impairment (%)		4 (1.9)	-
Mild liver disease (%)		1 (0.5)	-
Leukemia/Lymphoma (%)		1 (0.5)	-
HIV infection (%)		1 (0.5)	-
Home medications			
Cardiovascular (%)		140 (67.6)	-
Antiaggregant/anticoagulant (%)		52 (25.1)	-
Antidiabetic (%)		28 (13.5)	-
Psychotropic (%)		19 (9.2)	-
Thyroid (%)		15 (7.2)	-
Medication class burden	None (%)	50 (24.1)	-
	One (%)	80 (38.6)	-
	Multiclass (%)	77 (37.2)	-
CCI score (points)		4.0 (2.0–5.0)	0.0–11.0
CCI comorbidity (points)		2.0 (1.0–3.0)	0.0–8.0
ASA class	I (%)	10 (4.8)	-
	II (%)	136 (65.7)	-
	III (%)	61 (29.5)	-
Surgical and intraoperative management			
Operated organ	Prostate (%)	71 (34.3)	-
	Kidney (%)	35 (16.9)	-
	Uterus (%)	25 (12.1)	-
	Colon-rectum (%)	22 (10.6)	-
	Abdominal wall (%)	21 (10.1)	-
	Gallbladder (%)	18 (8.7)	-
	Other (%)	15 (7.2)	-
Surgical site	Pelvic (%)	98 (47.3)	-
	Retroperitoneal (%)	38 (18.3)	-
	Upper abdominal (%)	37 (17.9)	-
	Abdominal wall (%)	21 (10.1)	-
	Lower abdominal (%)	13 (6.3)	-
Intraoperative Trendelenburg (%)		119 (57.5)	-
Surgical time (min)		210.0 (162.5–250.0)	50.0–584.0
Anesthesia time (min)		240.0 (195.0–280.0)	85.0–625.0
Subarachnoid analgesia (%)		172 (83.1)	-
Intrathecal morphine (%)		172 (83.1)	-
Intrathecal Levobupivacaine	No	78 (37.7)	-
	5.0 mg	79 (38.2)	-
	10.0 mg	50 (24.1)	-
Intrathecal sufentanil (%)		86 (41.5)	-
Opioid induction	Fentanyl (%)	114 (55.1)	-
	Sufentanil (%)	93 (44.9)	-
Fentanyl equivalent dose induction (mcg/kg)		1.4 (1.2–1.8)	0.5–4.3
Propofol TCI (%)		14 (6.8)	-
MgSO_4_ administration (%)		111 (53.6)	-
Clonidine administration (%)		24 (11.6)	-
Intraoperative remifentanil infusion (%)		152 (73.4)	-
Model-derived intraoperative opioid AUC (ng⋅h/mL)		25.87 (19.73–36.04)	5.88–79.97
Intraoperative fluid management			
Colloid administration (%)		16 (7.7)	-
PRBC administration (%)		2 (1.0)	-
Standardized fluid administration (mL/kg/h)		5.37 (3.96–6.59)	0.46–16.2
Standardized urine output (mL/kg/h)		0.74 (0.49–1.10)	0.1–2.77
Standardized blood loss (mL/kg/h)		0.58 (0.42–0.79)	0.1–8.33
Standardized fluid output (mL/kg/h)		1.77 (1.44–2.22)	0.8–7.8
Standardized fluid balance (mL/kg/h)		3.38 (2.31–4.35)	−0.7–10.7

Continuous variables are reported as the median (q_1_–q_3_) and range (minimum–maximum); categorical variables are reported as a number (%). Standardized intraoperative variables are normalized by body weight and anesthesia duration, except blood loss, which is standardized by body weight and surgical duration.

**Table 2:** Baseline characteristics and perioperative management according to intrathecal analgesic regimen.

Variable		No-SA (*n* = 35)	IM (*n* = 43)	IM + L5 (*n* = 43)	IM + L5 + S3 (*n* = 36)	IM + L10 + S3 (*n* = 50)	*p*-Value
Age (years)		63.0 (49.0–70.5)	67.0 (62.0–71.0)	67.0 (56.0–73.0)	65.0 (56.7–70.5)	65.0 (51.2–71.0)	0.563
Sex, male (%)		21 (60.0)	30 (69.8)	26 (60.5)	22 (61.1)	30 (60.0)	0.862
BMI (kg/m^2^)		26.2 (24.3–30.7)	27.5 (25.3–30.8)	26.4 (24.5–29.0)	24.4 (23.1–28.6)	26.2 (24.2–30.4)	0.116
Comorbidities							
Solid Tumor	No (%)	18 (51.4)	9 (20.9)	10 (23.2)	15 (41.7)	10 (20.0)	0.008
	Localized (%)	16 (45.7)	32 (74.4)	33 (76.7)	21 (58.3)	40 (80.0)	
	Metastatic (%)	1 (2.8)	2 (4.6)	0 (0.0)	0 (0.0)	0 (0.0)	
Hypertension (%)		21 (60.0)	28 (65.1)	28 (65.1)	23 (63.9)	30 (60.0)	0.973
Smoking	No (%)	16 (45.7)	24 (55.8)	22 (51.2)	16 (44.4)	33 (66.0)	0.319
	Former (%)	10 (28.6)	9 (20.9)	7 (16.3)	12 (33.3)	10 (20.0	
	Current (%)	9 (25.7)	10 (23.2)	14 (32.5)	8 (22.2)	7 (14.0)	
Obesity (%)		12 (34.3)	15 (34.5)	7 (16.3)	5 (13.9)	13 (26.0)	0.093
COPD (%)		8 (22.8)	6 (13.9)	5 (11.6)	5 (13.9)	15 (30.0)	0.128
Diabetes	No (%)	33 (94.3)	37 (86.0)	37 (86.0)	34 (94.4)	40 (80.0)	0.214
	Uncomplicated (%)	1 (2.8)	5 (11.6)	6 (13.9)	2 (5.5)	10 (20.0)	
	End-organ injury (%)	1 (2.8)	1 (2.3)	0 (0.0)	0 (0.0)	0 (0.0)	
Myocardial infarction (%)		5 (14.3)	2 (4.6)	2 (4.6)	1 (2.8)	3 (6.0)	0.292
Atrial fibrillation (%)		1 (2.8)	3 (7.0)	1 (2.3)	3 (8.3)	1 (2.0)	0.499
Autoimmune disease (%)		1 (2.8)	0 (0.0)	2 (4.6)	2 (5.5)	3 (6.0)	0.594
CKD (%) *		1 (2.8)	1 (2.3)	0 (0.0)	1 (2.8)	1 (2.0)	0.912
PVD (%) *		0 (0.0)	1 (2.3)	1 (2.3)	0 (0.0)	2 (4.0)	0.807
CVA (%) *		0 (0.0)	1 (2.3)	0 (0.0)	1 (2.8)	2 (4.0)	0.725
Mild cognitive impairment (%) *		2 (5.7)	1 (2.3)	0 (0.0)	0 (0.0)	1 (2.0)	0.406
Mild liver disease (%) *		0 (0.0)	0 (0.0)	0 (0.0)	0 (0.0)	1 (2.0)	1.000
Leukemia/Lymphoma (%) *		0 (0.0)	0 (0.0)	0 (0.0)	1 (2.8)	0 (0.0)	0.343
HIV infection (%) *		0 (0.0)	0 (0.0)	0 (0.0)	1 (2.8)	0 (0.0)	0.343
Home medications							
Cardiovascular (%)		26 (74.3)	30 (69.8)	26 (60.5)	24 (66.7)	34 (68.0)	0.768
Antiaggregant/anticoagulant (%)		8 (22.8)	12 (27.9)	6 (13.9)	10 (27.8)	16 (32.0)	0.341
Antidiabetic (%)		2 (5.7)	7 (16.3)	7 (16.3)	2 (5.5)	10 (20.0)	0.190
Psychotropic (%)		7 (20.0)	3 (7.0)	3 (7.0)	4 (11.1)	2 (4.0)	0.126
Thyroid (%)		3 (8.6)	2 (4.6)	1 (2.3)	2 (5.5)	7 (14.0)	0.230
Medication class burden	None (%)	8 (22.8)	7 (16.3)	16 (37.2)	9 (25.0)	10 (20.0)	0.466
	One (%)	11 (31.4)	20 (46.5)	15 (34.9)	14 (38.9)	20 (40.0)	
	Multiclass (%)	16 (45.7)	16 (37.2)	12 (27.9)	13 (36.1)	20 (40.0)	
CCI score (points)		3.0 (1.0–5.0)	4.0 (3.0–5.0)	4.0 (3.0–5.5)	3.5 (2.0–5.0)	5.0 (3.0–6.0)	0.174
CCI comorbidity (points)		2.0 (0.0–3.0)	2.0 (2.0–2.5)	2.0 (2.0–2.0)	2.0 (0.0–2.2)	2.0 (2.0–3.0)	0.046
ASA class	I (%)	3 (8.6)	0 (0.0)	1 (2.3)	5 (13.9)	1 (2.0)	0.067
	II (%)	20 (57.1)	32 (74.4)	31 (72.1)	23 (63.9)	30 (60.0)	
	III (%)	11 (34.3)	11 (25.6)	11 (25.6)	8 (22.2)	19 (38.0)	
Surgical and intraoperative management							
Operated organ	Prostate (%)	9 (25.7)	15 (34.9)	18 (41.9)	11 (30.5)	18 (36.0)	0.021
	Kidney (%)	7 (20.0)	6 (13.9)	6 (13.9)	6 (16.7)	10 (20.0)	
	Uterus (%)	1 (2.8)	4 (9.3)	9 (20.9)	5 (13.9)	6 (12.0)	
	Colon-rectum (%)	1 (2.8)	8 (18.6)	5 (11.6)	3 (8.3)	5 (10.0)	
	Abdominal wall (%)	7 (20.0)	5 (11.6)	2 (4.6)	1 (2.8)	6 (12.0)	
	Gallbladder (%)	8 (22.8)	1 (2.3)	1 (2.3)	7 (19.4)	1 (2.0)	
	Other (%)	2 (5.7)	4 (9.3)	2 (4.6)	3 (8.3)	4 (8.0)	
Surgical site	Pelvic (%)	10 (28.6)	19 (44.2)	27 (62.8)	17 (47.2)	25 (50.0)	0.206
	Retroperitoneal (%)	8 (22.8)	7 (16.3)	6 (13.9)	6 (16.7)	11 (22.0)	
	Upper abdominal (%)	9 (25.7)	8 (18.6)	5 (11.6)	10 (27.8)	5 (10.0)	
	Abdominal wall (%)	7 (20.0)	5 (11.6)	2 (4.6)	1 (2.8)	6 (12.0)	
	Lower abdominal (%)	1 (2.8)	4 (9.3)	3 (7.0)	2 (5.5)	3 (6.0)	
Intraoperative Trendelenburg (%)		11 (31.4)	27 (62.8)	32 (74.4)	20 (55.5)	29 (58.0)	0.004
Surgical time (min)		165.0 (132.5–188.0)	225.0 (202.5–300.0)	220.0 (199.0–260.0)	194.0 (125.2–247.8)	210.0 (176.2–238.8)	<0.001
Anesthesia time (min)		193.0 (147.5–216.5)	250.0 (217.5–327.5)	250.0 (220.0–295.0)	224.0 (150.8–282.5)	245.0 (216.2–270.0)	<0.001
Opioid induction	Fentanyl (%)	28 (80.0)	43 (100.0)	43 (100.0)	0 (0.0)	0 (0.0)	<0.001
	Sufentanil (%)	7 (20.0)	0 (0.0)	0 (0.0)	36 (100.0)	50 (100.0)	
Fentanyl equivalent dose induction (mcg/kg)		1.5 (1.2–2.0)	1.3 (1.1–1.5)	1.6 (1.3–2.2)	1.5 (1.3–1.8)	1.3 (1.1–1.8)	0.009
Propofol TCI (%)		5 (14.3)	3 (7.0)	3 (7.0)	2 (5.5)	1 (2.0)	0.284
MgSO_4_ administration (%)		13 (37.1)	0 (0.0)	23 (53.5)	34 (94.4)	41 (82.0)	<0.001
Clonidine administration (%)		5 (14.3)	0 (0.0)	9 (20.9)	10 (27.8)	0 (0.0)	<0.001
Intraoperative remifentanil infusion (%)		34 (97.1)	43 (100.0)	42 (97.7)	28 (77.8)	5 (10.0)	<0.001
Model-derived intraoperative opioid AUC (ng⋅h/mL)		34.60 (27.42–43.80)	38.28 (26.79–44.80)	26.73 (19.38–30.31)	24.45 (20.87–37.96)	16.90 (13.94–22.23)	<0.001
Intraoperative fluid management							
Colloid administration (%)		3 (8.6)	0 (0.0)	6 (13.9)	7 (19.4)	0 (0.0)	0.002
PRBC administration (%)		0 (0.0)	0 (0.0)	1 (2.3)	1 (2.8)	0 (0.0)	0.507
Standardized fluid administration (mL/kg/h)		5.99 (5.00–7.66)	5.39 (4.23–6.28)	4.00 (3.36–5.68)	5.91 (4.91–8.43)	5.20 (3.68–6.47)	<0.001
Standardized urine output (mL/kg/h)		0.80 (0.62–1.17)	0.78 (0.49–1.44)	0.74 (0.39–1.10)	0.70 (0.47–0.93)	0.72 (0.49–1.03)	0.354
Standardized blood loss (mL/kg/h)		0.57 (0.45–0.74)	0.54 (0.36–0.65)	0.60 (0.39–0.72)	0.61 (0.46–0.88)	0.61 (0.46–0.87)	0.310
Standardized fluid output (mL/kg/h)		1.75 (1.52–2.27)	1.84 (1.43–2.33)	1.80 (1.38–2.25)	1.74 (1.42–2.13)	1.79 (1.41–2.11)	0.899
Standardized fluid balance (mL/kg/h)		4.05 (2.93–5.93)	3.32 (2.61–4.19)	2.47 (1.66–3.81)	3.99 (3.19–6.66)	3.16 (2.40–4.07)	<0.001

Continuous variables are presented as the median (q1–q3) and were compared using the Kruskal–Wallis test. Categorical variables are presented as numbers (%) and were compared using the χ^2^ test or Fisher’s exact test (*) for variables with fewer than five observations overall. Intraoperative blood loss was standardized by weight and surgical duration, and otherwise by weight and anesthesia duration. Abbreviations: No-SA, no subarachnoid analgesia; IM, intrathecal morphine; IM + L5, intrathecal morphine plus 5 mg levobupivacaine; IM + L5 + S3, intrathecal morphine plus 5 mg levobupivacaine and 3 μg sufentanil; IM + L10 + S3, intrathecal morphine plus 10 mg levobupivacaine and 3 μg sufentanil.

**Table 3:** Univariate and multivariable Gamma regression models evaluating factors associated with model-derived intraoperative opioid exposure (AUC/h) and standardized intraoperative fluid administration.

Variable		Model AUC/h				Model Standardized Intraoperative Fluid Administration			
		Univariate		Multivariate Model		Univariate		Multivariate Model	
		β% (95% CI)	*p*-Value	β% (95% CI)	*p*-Value	β% (95% CI)	*p*-Value	β% (95% CI)	*p*-Value
Age		−0.6 (−1.1 to −0.1)	0.014	−0.5 (−0.9 to −0.1)	0.021	−0.4 (−0.8 to 0.0)	0.048	+0.1 (−0.4 to +0.5)	0.782
Sex, male		−8.3 (−19.7 to +4.6)	0.197	−2.5 (−11.1 to +6.8)	0.583	−16.3 (−25.1 to −6.5)	0.002	−3.0 (−12.7 to +7.7)	0.567
BMI		−1.6 (−2.9 to −0.2)	0.022	−0.2 (−1.1 to +0.8)	0.712	−3.5 (−4.5 to −2.4)	<0.001	−2.9 (−4.0 to −1.8)	<0.001
Medication class burden	None	Ref.	0.280	Ref.	0.141	Ref.	0.172	Ref.	0.582
	One	−11.0 (−24.7 to +5.0)		+1.2 (−9.6 to +13.2)		−6.4 (−18.8 to +7.7)		+4.6 (−8.0 to +18.7)	
	Multiclass	−11.7 (−25.4 to +4.3)		−8.7 (−20.0 to +4.3)		−12.6 (−24.3 to +0.7)		−1.2 (−15.3 to +15.1)	
Smoking status	No	Ref.	<0.001	Ref.	0.002	Ref.	0.792	Ref.	0.784
	Former	−5.5 (−18.7 to +10.2)		−3.3 (−12.6 to +7.0)		−1.0 (−13.7 to +13.9)		+3.3 (−7.8 to +15.9)	
	Current	+35.3 (+16.4 to +57.7)		+17.2 (+6.2 to +29.5)		+4.3 (−9.1 to +19.9)		+3.2 (−7.9 to +15.8)	
CCI comorbidity		−2.2 (−5.9 to +1.8)	0.277	+3.5 (−0.5 to +7.8)	0.087	−5.4 (−8.4 to −2.3)	<0.001	−7.0 (−11.1 to −2.6)	0.002
ASA class	I	Ref.	0.160	Ref.	0.580	Ref.	0.010	Ref.	0.120
	II	−18.7 (−40.9 to +8.9)		−10.0 (−28.1 to +12.0)		−27.3 (−43.8 to −7.6)		+12.5 (−12.9 to +44.1)	
	III	−25.1 (−46.1 to +1.8)		−8.0 (−29.5 to +19.6)		−31.7 (−47.7 to −12.2)		+29.0 (−4.7 to +73.8)	
Surgical site	Pelvic	Ref.	0.272	Ref.	0.521	Ref.	0.016	Ref.	0.148
	Retroperitoneal	+18.8 (−0.1 to +42.1)		+14.5 (−9.8 to +45.7)		+12.8 (−2.8 to +31.3)		+14.2 (−13.2 to +50.9)	
	Upper abdominal	+15.6 (−3.0 to +38.5)		+4.5 (−13.3 to +26.7)		+29.7 (+11.6 to +51.2)		+24.6 (+0.5 to +55.8)	
	Abdominal wall	+9.0 (−12.2 to +36.8)		+12.7 (−11.7 to +44.4)		+6.5 (−11.5 to +29.2)		+9.9 (−16.9 to +46.1)	
	Lower abdominal	+13.9 (−12.5 to +51.0)		+11.3 (−5.5 to +31.9)		+9.3 (−12.8 to +38.9)		+13.6 (−6.0 to +38.1)	
Intraoperative Trendelenburg		−8.6 (−19.8 to +4.1)	0.175	+13.0 (−8.4 to +39.7)	0.254	−10.9 (−20.3 to −0.4)	0.043	+12.1 (−12.0 to +43.4)	0.357
Intrathecal regimen	No-SA	Ref.	<0.001	Ref.	<0.001	Ref.	<0.001	Ref.	<0.001
	IM	−2.2 (−18.2 to +16.8)		+14.0 (−0.9 to +31.1)		−18.1 (−30.4 to −3.7)		−8.5 (−22.1 to +7.3)	
	IM + L5	−28.7 (−40.4 to −14.9)		−36.4 (−45.0 to −26.6)		−28.9 (−39.6 to −16.3)		−24.9 (−36.3 to −11.5)	
	IM + L5 + S3	−30.2 (−42.0 to −15.9)		−24.3 (−40.6 to −4.5)		+7.3 (−9.5 to +27.0)		−17.3 (−37.4 to +8.0)	
	IM + L10 + S3	−50.9 (−58.7 to −41.7)		−44.8 (−56.3 to −31.0)		−19.7 (−31.4 to −6.1)		−31.0 (−47.1 to −11.1)	
Opioid induction, Fentanyl		+43.7 (+27.3 to +62.2)	<0.001	+6.0 (−18.3 to +36.5)	0.654	−13.1 (−22.0 to −3.2)	0.011	−17.4 (−38.4 to +9.6)	0.187
Fentanyl-equivalent induction dose		+39.3 (+25.1 to +55.5)	<0.001	+47.5 (+35.9 to +60.4)	<0.001	+12.4 (+1.8 to +24.5)	0.021	+0.2 (−8.6 to +10.2)	0.969
Propofol TCI, yes		+38.3 (+8.1 to +80.3)	0.009	+40.2 (+19.4 to +65.3)	<0.001	−3.5 (−22.1 to +21.3)	0.755	−4.3 (−20.3 to +15.7)	0.647
MgSO_4_ administration, yes		−16.0 (−26.1 to −4.5)	0.007	+0.1 (−11.8 to +13.4)	0.992	+5.1 (−5.9 to +17.4)	0.378	+1.9 (−11.6 to +17.2)	0.798
Clonidine administration, yes		+28.0 (+5.3 to +57.4)	0.013	+20.0 (+4.9 to +37.5)	0.008	+2.4 (−13.6 to +22.3)	0.787	−2.6 (−16.5 to +13.8)	0.735
Intraoperative standardized blood loss		+2.0 (−7.2 to +14.1)	0.704	+1.9 (−4.4 to +9.3)	0.576	+24.8 (+14.4 to +37.8)	<0.001	+18.1 (+9.2 to +28.9)	<0.001

Results are reported as percentage change (β%) with 95% confidence intervals (95% CIs) derived from exponentiated regression coefficients. For categorical predictors, *p*-values correspond to the overall likelihood ratio test for the variable. Continuous intraoperative variables were standardized by body weight and anesthesia duration unless otherwise specified. The fentanyl-equivalent induction dose is a constituent of the AUC/h outcome; its coefficient is structural (part–whole) and is not interpretable as an independent clinical association.

**Table 4:** Univariate and multivariable logistic regression analyses evaluating factors associated with a pain-free postoperative course (NRS = 0 throughout the first 48 postoperative hours).

Variable		Pain-Free Model			
		Univariate		Multivariate Model	
		OR (95% CI)	*p*-Value	OR (95% CI)	*p*-Value
Age		1.04 (1.01–1.06)	0.007	1.02 (0.98–1.06)	0.255
Sex, male		1.57 (0.82–3.02)	0.170	0.97 (0.43–2.16)	0.932
BMI		1.03 (0.96–1.11)	0.373	1.04 (0.96–1.13)	0.346
Medication class burden	None	Ref.	0.736	Ref.	0.324
	One	1.03 (0.46–2.32)		0.53 (0.18–1.54)	
	Multiclass	1.32 (0.58–3.02)		0.39 (0.11–1.33)	
Smoking	None	Ref.	0.507	Ref.	0.937
	Former	1.50 (0.68–3.37)		1.13 (0.45–2.90)	
	Current	0.90 (0.42–1.96)		0.93 (0.39–2.23)	
CCI comorbidity		1.49 (1.19–1.90)	<0.001	1.52 (1.03–2.32)	0.034
ASA class	I	Ref.	0.159	Ref.	0.982
	II	2.00 (0.50–9.87)		0.87 (0.15–5.73)	
	III	3.41 (0.79–17.87)		0.81 (0.09–7.66)	
Surgical site	Pelvic	Ref.	0.017	Ref.	0.499
	Retroperitoneal	0.37 (0.16–0.86)		0.43 (0.16–1.11)	
	Upper abdominal	0.48 (0.18–1.29)		0.91 (0.29–2.93)	
	Abdominal wall	0.18 (0.04–0.63)		0.57 (0.11–2.51)	
	Lower abdominal	1.19 (0.29–5.92)		0.82 (0.17–4.64)	
Subarachnoid analgesia, yes		4.11 (1.61–11.92)	0.003	3.33 (1.06–11.79)	0.040
Postoperative elastomeric pump, yes		1.09 (0.51–2.38)	0.824	1.26 (0.49–3.40)	0.640

Results are reported as odds ratios (ORs) with 95% confidence intervals (95% CIs). For categorical predictors, *p*-values correspond to the overall likelihood ratio test for the variable.

## Data Availability

The data presented in this study are available on request from the corresponding author. Data sharing is subject to applicable ethical and privacy regulations.

## Supplementary Materials

The following supporting information can be downloaded at: https://www.mdpi.com/article/10.3390/clinpract16090167/s1.

## Abbreviations

The following abbreviations are used in this manuscript:

ANOVA	Analysis of Variance
ASA	American Society of Anesthesiologists (Physical Status)
AUC/h	Model-derived area under the concentration–time curve per hour
BIS	Bispectral Index
BMI	Body Mass Index
CCI	Charlson Comorbidity Index
CI	Confidence Interval
CKD	Chronic Kidney Disease
COPD	Chronic Obstructive Pulmonary Disease
CVA	Cerebro Vascular Accident
DHARMa	Diagnostics for HierArchical Regression Models (R package)
EMM	Estimated marginal mean
ERAS	Enhanced Recovery After Surgery
FDR	False discovery rate
GA	General anesthesia
HIV	Human Immunodeficiency Virus
IM	Intrathecal morphine (regimen)
IM + L5	Intrathecal morphine plus levobupivacaine 5 mg
IM + L5 + S3	Intrathecal morphine plus levobupivacaine 5 mg and sufentanil 3 µg
IM + L10 + S3	Intrathecal morphine plus levobupivacaine 10 mg and sufentanil 3 µg
IQR	Interquartile range
LA	Local anesthetic
MgSO_4_	Magnesium sulfate
MME	Morphine milligram equivalents
No-SA	No subarachnoid analgesia (regimen)
NRS	Numeric Rating Scale
OR	Odds ratio
PONV	Postoperative Nausea and Vomiting
PRBC	Packed red blood cell
PVD	Peripheral Vascular Disease
SA	Subarachnoid analgesia
SD	Standard deviation
STROBE	Strengthening the Reporting of Observational Studies in Epidemiology
TCI	Target-controlled infusion
VIF	Variance inflation factor
