# Supplementary material for: The Intrathecal Analgesia in Robotic Surgery (IARS) Study: Real-World Associations with Intraoperative Opioid Exposure, Fluid Administration, and Postoperative Pain-Free Recovery
Source: Clin Pract. 2026 Sep 9;16(9):167. doi: 10.3390/clinpract16090167 (PMC13605514; doi:10.3390/clinpract16090167)
Supplement: Supplementary file 1 [file clinpract-16-00167-s001.zip › clinpract-4454757-supplementary.pdf]

SUPPLEMENTARY FILE

1. Materials and Methods

1.1 Patient's home therapy

Medication history was collected from the medical records, and major therapeutic classes were categorized as follows:

a) Antiplatelet/anticoagulant therapy: cyclooxygenase-1 (COX-1) inhibitors, primarily acetylsalicylic acid; P2Y12 receptor inhibitors such as clopidogrel, prasugrel, and ticagrelor (and, when applicable, ticlopidine); phosphodiesterase inhibitors/vascular-acting antiplatelet agents (dipyridamole and cilostazol). Anticoagulant therapy included vitamin K antagonists (warfarin); low-molecular-weight heparins (e.g., enoxaparin, dalteparin, nadroparin, tinzaparin), and fondaparinux; and direct oral anticoagulants (DOACs/NOACs), comprising direct factor Xa inhibitors (apixaban, rivaroxaban, edoxaban) and the direct thrombin inhibitor dabigatran.

b) Cardiovascular drugs: beta-blockers (e.g., metoprolol, bisoprolol, carvedilol, atenolol, nebivolol); angiotensin-converting enzyme inhibitors and angiotensin II receptor blockers (ARBs) (e.g., ramipril, enalapril, lisinopril; losartan, valsartan, candesartan, olmesartan); calcium-channel blockers (dihydropyridine and non-dihydropyridine: amlodipine, nifedipine, felodipine; verapamil, diltiazem); diuretics (thiazide and thiazide-like: hydrochlorothiazide, indapamide; loop diuretics: furosemide, torasemide; potassium-sparing/anti-aldosterone agents: spironolactone, eplerenone; amiloride); antianginal agents (nitrates: nitroglycerin/isosorbide; ranolazine; ivabradine); centrally acting alpha-2 agonists and other sympatholytics (clonidine, methyldopa, moxonidine); alpha-blockers (doxazosin, terazosin, prazosin; tamsulosin); and antiarrhythmics (flecainide, propafenone, amiodarone).

c) Psychoactive drugs: benzodiazepines (e.g., diazepam, lorazepam, alprazolam, clonazepam, delorazepam, lormetazepam, bromazepam); antidepressants, including SSRIs (sertraline, citalopram/escitalopram, paroxetine, fluoxetine), SNRIs (venlafaxine, duloxetine), tricyclics (amitriptyline, clomipramine, nortriptyline), NaSSAs (mirtazapine), and other antidepressants (bupropion, trazodone); and antipsychotics, categorized as typical (haloperidol, chlorpromazine) and atypical (quetiapine, olanzapine, risperidone, aripiprazole, clozapine).

d) Endocrine therapy (thyroid hormones): levothyroxine and liothyronine.

e) Antidiabetic medications: any ongoing chronic antidiabetic therapy was recorded, distinguishing between insulin and non-insulin agents. Non-insulin antidiabetic drugs included biguanides (metformin); sulfonylureas (gliclazide, glimepiride, glibenclamide); meglitinides/glinides (repaglinide); DPP-4 inhibitors (sitagliptin, linagliptin, saxagliptin, vildagliptin); GLP-1 receptor agonists (liraglutide, semaglutide, dulaglutide, exenatide); SGLT2 inhibitors (empagliflozin, dapagliflozin, canagliflozin); thiazolidinediones (pioglitazone); and, when present, other less commonly used agents (acarbose).

Medication class burden was defined as the number of chronic medication classes the patient was taking at the time of the preoperative assessment, counted across prespecified categories. For each category, a dichotomous variable (present/absent) was assigned, and the burden was computed as the sum of present categories, regardless of the number of individual drugs within each category. For analytical purposes, medication class burden was additionally categorized into ordinal levels (none, one, multiclass).

1.2 Intrathecal drugs preparation

Intrathecal drugs were prepared under aseptic conditions immediately before administration, using dedicated sterile syringes, avoiding unnecessary admixtures, and ensuring batch

traceability in accordance with internal procedures. All dilutions were performed with 0.9% sodium chloride (normal saline) drawn aseptically.

Morphine was diluted under sterile conditions (one 1.0 mL ampoule containing 10.0 mg was diluted to a final volume of 10.0 or 20.0 mL, yielding a final concentration of 1.0 or 0.5 mg/mL). The volume corresponding to a dose of at least 100 mcg was then withdrawn using an insulin syringe.

Levobupivacaine was prepared to obtain a final concentration of 0.33%; operationally, 10.0 mg corresponds to 2.0 mL of 0.5% levobupivacaine and 5.0 mg to 1.0 mL, with the addition of diluent to reach the target final concentration (respectively, 1.0 mL or 0.5 mL of 0.9% sodium chloride).

Sufentanil was drawn from an ampoule (1.0 mL, 50.0 mcg/mL) and diluted under sterile conditions to achieve a concentration of 5.0 or 10.0 mcg/mL. The volume equivalent to a 3.0 mcg dose was then aspirated into a dedicated insulin syringe (0.6 mL or 0.3 mL depending on the dilution).

After preparation, the solutions were administered intrathecally as separate injections. At the end of the spinal analgesia, the puncture site was dressed, and the patient was returned to the supine position.

1.3 Single shot subarachnoid analgesia

For patients who provided specific consent, single-shot SA was performed before induction of GA, with the patient in the sitting position on the operating table under sterile conditions (27-gauge Whitacre needle, midline approach). The interspace was selected based on the surgical site: a higher lumbar interspace (L_2-L_3) was preferred for upper abdominal procedures, whereas a lower interspace (L_3-L_4 or L_4-L_5) was used for lower abdominal or pelvic surgery. Free cerebrospinal fluid flow confirmed correct intrathecal placement. The intrathecal regimen was determined by the attending anesthesiologist on a case-by-case

basis. All patients receiving SA received intrathecal morphine (minimum 100 mcg). At the anesthesiologist's discretion, levobupivacaine 0.33% (5.0 or 10.0 mg) and/or sufentanil (3.0 mcg) were added. Consequently, the administered SA regimens ranged from intrathecal morphine alone to morphine combined with levobupivacaine and sufentanil in various combinations. Drug doses were documented in the anesthesia chart;

1.4 Standardized perioperative protocol

1.4.1 Premedication and prophylaxis

Upon entering the operating room, patients received two large-bore intravenous (IV) lines. Before induction, the following agents were administered: antibiotic prophylaxis with cefazolin 2.0 g IV (plus metronidazole 500.0 mg IV when appropriate, based on surgical indication); esomeprazole 40.0 mg IV for stress ulcer prophylaxis; and dexamethasone 4.0 mg IV for PONV prevention.

1.4.2 Monitoring

Standard monitoring included continuous pulse oximetry (SpO_2), a 5-lead electrocardiogram (ECG), and noninvasive arterial blood pressure measurement. When indicated by procedural complexity or patient comorbidities, an arterial line was placed after induction for continuous invasive blood pressure monitoring. Bispectral index (BIS; Medtronic, Dublin, Ireland) guided anesthetic depth targeting. Neuromuscular blockade was monitored using train-of-four (TOF) stimulation (TOFscan; Dräger, Lübeck, Germany). Core temperature (pharyngeal probe) and urine output were continuously monitored and recorded.

1.4.3 Ventilatory management

After orotracheal intubation (performed when TOF = 0), controlled ventilation was initiated using a lung-protective strategy. Throughout the procedure, including periods of pneumoperitoneum and positional changes (e.g., Trendelenburg), ventilator settings were adjusted to maintain a driving pressure <15 cmH_2O and an end-tidal CO_2 ($EtCO_2$) of 35-40 mmHg. Pneumoperitoneal pressure was maintained at <13.0 mmHg throughout the procedure.

1.4.4 Hemodynamic management.

A continuous norepinephrine infusion was initiated if the mean arterial pressure (MAP) remained <65 mmHg despite initial volume optimization, and the infusion was titrated to the hemodynamic target. If hypertension (MAP >100 mmHg or systolic blood pressure >160 mmHg) persisted for ≥10 minutes despite adequate anesthetic depth and remifentanil titration, clonidine was administered in IV boluses of 1.0 mcg/kg (maximum cumulative dose 150.0 mcg). For persistent tachycardia (heart rate >100 bpm for ≥10 minutes), despite optimization of anesthetic depth and analgesia and correction of reversible causes, metoprolol was administered as a slow IV bolus (2.5–5.0 mg over 1–2 minutes).

1.4.5 Fluid and temperature management.

A baseline crystalloid infusion was maintained at 1-3 mL/kg/h and adjusted to clinical targets and surgical phase. Active warming was provided throughout using a forced-air warming mattress.

1.4.6 Neuromuscular blockade protocol

Neuromuscular blockade was monitored continuously with TOFscan. If the post-tetanic count (PTC) exceeded 3, a rocuronium bolus (0.3 mg/kg IV) was administered to deepen the block, as per institutional protocol.

1.4.7 Pre-emergence multimodal analgesia

At specimen extraction, a standardized multimodal analgesic combination was administered: paracetamol 1.0 g IV, ketorolac 30 mg IV when not contraindicated, and tramadol 1.0 mg/kg IV (max 100.0 mg). Trocar sites were infiltrated with 0.5% ropivacaine (total volume 20 mL, distributed across all trocar sites) by the surgeon. Ondansetron 4 mg IV was administered before awakening for PONV prophylaxis.

1.4.8 Emergence and extubation.

At the end of surgery, the remifentanil infusion was discontinued, and the neuromuscular block was reversed with sugammadex IV, titrated per institutional protocol until a TOF ratio >0.90 was achieved. Hypnotic agents were discontinued, and the patient was extubated when clinical criteria were met (adequate spontaneous ventilation, satisfactory oxygenation, responsiveness, and protective airway reflexes). The patient was then transferred to the recovery area.

1.4.9 Intraoperative fluid balance variables

Intraoperative fluid administration was defined as the total volume of crystalloids and colloids infused from the start of anesthesia induction to extubation, including the volumes used as vehicles for drug administration (drug flushes and dilutions). Colloid administration was recorded separately as a binary variable. Estimated intraoperative blood loss was derived from calibrated suction canister volumes and weighed surgical sponges, after subtraction of irrigation fluids, as documented by the attending anesthesiologist and the circulating nurse in the operative record. Urine output was measured from an indwelling bladder catheterization performed after induction. Total fluid output was defined as the sum of urine output, estimated blood loss, and perspiratio insensibilis (estimated as 0.5 mL/kg/h); intraoperative fluid balance was computed as the difference between total fluid input

(crystalloids + colloids + transfusion products) and total fluid output. All volume variables were normalized to actual body weight and anesthesia duration, except for blood loss, which was standardized for surgical time, yielding values expressed in mL/kg/h.

1.5 Intraoperative remifentanil infusion protocol

Remifentanil was administered in TCI mode (Minto effect-site model), with effect-site concentration (C_e) titrated to hemodynamic responses. Remifentanil infusion was initiated at the attending anesthesiologist's discretion, typically at skin incision or upon the onset of hemodynamic responses to surgical stimulation (starting C_e 0.5 ng/mL).

The attending anesthesiologist titrated remifentanil according to a standardized protocol, with clinical discretion permitted for patient safety:

- If mean arterial pressure (MAP) decreased >20% from baseline or <65 mmHg with BIS 40-60: reduce C_e by 0.5 ng/mL; treat hypotension with crystalloid or colloid (succinylated gelatin; Gelofusine®) bolus 250 mL and/or ephedrine (5.0 mg IV).
- If heart rate (HR) <45 bpm: reduce C_e by 0.5 ng/mL; administer atropine (0.5 mg IV).
- If hypertension (defined as MAP >100 mmHg or systolic blood pressure >160 mmHg) or tachycardia (defined as HR >100 bpm) occurred with BIS 40-60: increase C_e by 0.5 ng/mL (maximum 5.0 ng/mL) and reassess after 2 minutes.
- If BIS >60 with stable hemodynamics, deepen the hypnotic (sevoflurane/propofol) first.
- If BIS <40 with stable hemodynamics, lighten the hypnotic; consider reducing remifentanil by 0.3-0.5 ng/mL.

1.6 Procedures classifications

Procedures were categorized according to the operated organ and anatomical location as:

- Pelvic surgery: procedures primarily performed within the pelvis (e.g., prostate, bladder, gynecologic pelvic procedures, rectal surgery including total mesorectal excision/low anterior resection/abdominoperineal resection).
- Lower abdominal surgery (inframesocolic): procedures targeting organs below the transverse mesocolon, including left-sided colon surgery (distal transverse colon, splenic flexure, descending colon, sigmoid colon).
- Upper abdominal surgery (supramesocolic): procedures targeting organs above the transverse mesocolon, including hepatobiliary surgery (e.g., cholecystectomy) and right-sided colon surgery (cecum, ascending colon, hepatic flexure, and proximal transverse colon).
- Retroperitoneal surgery: kidney/adrenal/ureter (e.g., nephrectomy, adrenalectomy, pyeloplasty).
- Abdominal wall surgery: ventral/incisional hernia repair (including umbilical/epigastric hernia).

1.7 Intraoperative opioid exposure computation (AUC/h)

Intraoperative opioid administration was recorded separately for induction and maintenance. Induction opioid doses were standardized to actual body weight and expressed as fentanyl equivalents (µg/kg); sufentanil doses were converted using a 10:1 fentanyl-equivalence factor. This conversion was applied solely to place different opioids on a common reporting scale and does not imply exact pharmacodynamic equivalence.

Remifentanil exposure was additionally reported using directly observed measures, including the absolute cumulative intraoperative dose (µg), the proportion of patients receiving a remifentanil infusion, and the remifentanil administration rate, a weight- and time-standardized measure (ng/kg/min) obtained by dividing the cumulative remifentanil dose by actual body weight and surgical duration. Note that this descriptive remifentanil-specific rate

is standardized by surgical duration, whereas the composite opioid administration rate used in the robustness analysis (see Section 1.9) is standardized by anesthesia duration, consistent with AUC/h.

Because fentanyl, sufentanil, and remifentanil have substantially different elimination kinetics, cumulative administered doses alone may not adequately reflect their relative contribution to intraoperative opioid exposure. Therefore, a model-derived area-under-the-concentration-time-curve metric (AUC) was used as the prespecified primary exposure measure. The purpose of this metric was not to estimate patients' true plasma concentrations, which were not measured, but to provide a standardized summary of cumulative intraoperative opioid exposure across heterogeneous opioid regimens while approximately accounting for differences in drug disposition.

Individual concentration-time profiles were reconstructed using a simplified one-compartment pharmacokinetic model with first-order elimination. Concentrations were estimated at predefined time points from induction until completion of surgical dressings, and the area under each estimated AUC was calculated using the trapezoidal rule [1,2]:

$$AUC = \sum_{i=0}^{n-1} \frac{(C_i + C_{i+1})}{2} \cdot (t_{i+1} - t_i)$$

where C_i and C_{i+1} are the estimated concentrations at successive time points t_i and t_{i+1}, and n is the total number of time points.

For fentanyl and sufentanil boluses, the estimated initial concentration after each administration was calculated as:

$$C_0 = \frac{D}{V_d}$$

where D is the bolus dose (expressed as µg), and V_d is the apparent volume of distribution (expressed as mL/kg). The subsequent decay was modeled as:

$$C_t = C_0 \cdot e^{-k_e \cdot t}$$

with

$$k_e = \frac{Cl}{V_d}$$

where Cl is clearance (expressed as mL/kg/min), and k_e is the first-order elimination constant.

Remifentanil was administered clinically using an effect-site target-controlled infusion according to the Minto model, with targets adjusted during surgery based on hemodynamic responses. Because the complete sequence of target changes and pump-delivered infusion rates was not consistently available in the electronic records, the observed cumulative remifentanil dose was represented as an equivalent constant infusion rate (ng/kg/min) over the recorded maintenance period, from skin incision to completion of surgical dressings. The corresponding estimated concentration profile was calculated as:

$$C_t = \frac{R}{Cl} \cdot (1 - e^{-k_e \cdot t})$$

where (R) represents the equivalent constant infusion rate derived from the observed cumulative remifentanil dose and the recorded infusion duration. This reconstruction, therefore, preserved the total administered dose but did not reproduce the short-term concentration fluctuations caused by individual target adjustments. For patients who did not receive remifentanil, the remifentanil component was assigned a true value of zero.

Representative adult population pharmacokinetic parameters were obtained from official product information. The following values were used: V_d 5,500 mL/kg and Cl 10 mL/kg/min for fentanyl; V_d 4,000 mL/kg and Cl 8 mL/kg/min for sufentanil; and V_d 350 mL/kg and Cl 60 mL/kg/min for remifentanil. When the source reported a range, a representative midpoint value was selected and applied uniformly to all patients.

To combine exposure from different opioids on a common reporting scale, sufentanil-derived AUC values were multiplied by 10, reflecting its approximately ten-fold greater potency relative to fentanyl. This conversion was used exclusively for exposure standardization and

should not be interpreted as an exact pharmacodynamic equivalence. Remifentanil exposure was combined with fentanyl-equivalent bolus exposure using the same prespecified operational scale.

For patients receiving more than one opioid, the standardized AUC contributions of the individual drugs were summed. This additive calculation was intended solely to quantify cumulative exposure and did not attempt to model pharmacodynamic interactions among opioids.

To facilitate comparison among procedures of different durations, the resulting total AUC was divided by the anesthesia duration and expressed as AUC per hour (AUC/h). Anesthesia duration was chosen because it encompasses the entire period during which opioid administration contributes to intraoperative exposure, including induction, which precedes skin incision. Standardizing by surgical duration would have excluded this early exposure despite its contribution to the cumulative AUC. AUC/h therefore represents a duration-standardized, model-derived index of overall intraoperative opioid exposure.

This approach relies on simplifying assumptions, including a one-compartment structure, fixed population pharmacokinetic parameters, constant clearance, absence of patient-specific pharmacokinetic variability, and representation of variable remifentanil TCI administration as an equivalent constant infusion. Accordingly, AUC/h should be interpreted as a comparative exposure index rather than as a measurement or precise prediction of individual plasma opioid concentrations.

To assess whether the principal findings depended on these pharmacokinetic assumptions, a complementary robustness analysis was performed using a directly observed cumulative opioid administration rate standardized to body weight and anesthesia duration. The model-derived AUC/h and the directly observed administration-rate analysis were therefore considered complementary measures addressing, respectively, pharmacokinetically standardized exposure and recorded drug administration.

1.8 Postoperative pain assessment and rescue protocol

Postoperative pain at rest was assessed by trained nursing staff using an 11-point Numeric Rating Scale (NRS, 0 = no pain, 10 = worst imaginable pain) at nine predefined time points during the first 48 postoperative hours: at awakening, 30 minutes, 1, 3, 6, 12, 24, 36, and 48 hours after extubation. Assessments were recorded in the institutional pain chart together with rescue drug administration and adverse events.

Postoperatively, all patients received paracetamol 1.0 g every 8 hours. At the anesthesiologist's discretion, additional opioid analgesia was prescribed as tramadol 50.0 mg every 12 hours or as a continuous infusion via an elastomeric pump (morphine or tramadol, 5.0 mL/h). Rescue analgesia for NRS >3 at rest consisted of ketorolac 30.0 mg IV; if NRS remained >7, tramadol 50.0 mg or morphine 3.0 mg IV was administered.

1.9 Detailed statistical modeling strategy

Continuous variables were summarized as mean ± standard deviation (SD) or median with first and third quartiles (q_1-q_3), according to the distribution assessed using the Shapiro-Wilk test. Categorical variables were reported as counts and percentages.

Associations between candidate predictors and the two primary continuous outcomes (AUC/h and standardized intraoperative fluid administration) were evaluated using univariate and multivariable generalized linear models (GLMs) with a Gamma distribution and log link. The Gamma family was selected because both outcomes were continuous, strictly positive, and right-skewed. The log link allowed model coefficients to be interpreted as proportional changes.

Two complementary multivariable models were fitted for each outcome. The first model included the intrathecal analgesic regimen as a single categorical variable, allowing comparisons between the different neuraxial strategies. The second model replaced the

regimen variable with its individual intrathecal components (morphine, levobupivacaine dose, and sufentanil), entered simultaneously as separate covariates to assess the independent association of each drug with the outcome after adjustment for the others.

The following predictors were evaluated in the multivariable models: age, sex, BMI, smoking status, medication class burden, CCI-comorbidity, ASA Physical Status, surgical site, intraoperative positioning, induction opioid type, fentanyl-equivalent induction dose, propofol TCI, $MgSO_4$ administration, clonidine administration, and standardized intraoperative blood loss.

Exponentiated coefficients (exp[β]) were reported as exposure ratios and expressed as percentage changes with corresponding 95% confidence intervals (95% CI). P-values were derived from likelihood-ratio tests (LRT) comparing nested models with and without the term of interest. For categorical predictors, p-values reflected the overall (global) effect.

Because AUC/h is a derived-model pharmacokinetic metric, robustness analyses were also conducted using a directly observable exposure variable: the total intraoperative opioid administration rate, standardized to body weight and anesthesia duration (ng/kg/min).

For this analysis, induction bolus doses and intraoperative infusion doses were summed, assuming operational dose equivalence between fentanyl and remifentanil. Although this assumption is pharmacologically approximate, given the distinct pharmacokinetic and pharmacodynamic properties of the two opioids, this complementary metric was intended to assess whether the direction and statistical significance of the principal findings remained consistent, independent of the AUC/h modeling assumptions.

For the postoperative pain-free endpoint, a complete-case approach was adopted, including only patients with NRS data available at all predefined postoperative time points.

Because missing postoperative pain assessments may not have occurred completely at random, baseline characteristics were compared between patients with complete and incomplete NRS datasets to evaluate potential systematic differences between groups.

Accordingly, the assumption of missing completely at random (MCAR) should be interpreted cautiously, and findings related to the postoperative pain-free endpoint should be considered exploratory and hypothesis-generating.

To account for potential clustering by anesthesia provider, all primary multivariable models were refitted as mixed-effects models, including the attending anesthesiologist as a random intercept.

For continuous outcomes (AUC/h and standardized intraoperative fluid administration), mixed-effects Gamma regression models with a log link were fitted. For the binary pain-free outcome, a mixed-effects logistic regression model was used.

Fixed-effect specifications remained unchanged compared with the primary analyses. Provider-adjusted models were interpreted as robustness analyses to assess whether the principal associations persisted after accounting for between-anesthesiologist variability.

Model diagnostics included simulation-based residual analyses using the DHARMa package and assessment of multicollinearity using variance inflation factors (VIF).

Residual diagnostics evaluated residual uniformity, dispersion, and potential outliers. Multicollinearity was assessed using VIF values, with values <5 considered acceptable.

All analyses were performed in R version 4.5.2 (R Foundation for Statistical Computing). The following packages were used: glmmTMB and/or lme4 for generalized linear and mixed-effects models with Gamma and binomial families; DHARMa for simulation-based residual diagnostics; car for the computation of adjusted variance inflation factors; ggplot2 and ggalluvial for figure generation.

2.Results

2.1 AUC/h model and intrathecal regimen

No evidence of severe multicollinearity was observed based on the adjusted VIF metric (all VIFs < 5). Overall, diagnostics suggested limited collinearity among most covariates, with several predictors showing adjusted values close to 1, consistent with negligible shared variance (e.g., smoking 1.10, TCI 1.11, intraoperative standardized blood loss 1.14, clonidine 1.17, sex 1.20, BMI 1.20, fentanyl-equivalent induction dose 1.24, medication class burden 1.24, and age 1.39). Most multi-level factor variables also showed low adjusted values, including ASA physical status (1.37) and surgical site (1.38).

A subset of predictors exhibited moderate adjusted values, indicating some shared variance with other model terms but remaining within acceptable ranges for multivariable clinical regression. These included intrathecal morphine 1.44, intrathecal levobupivacaine dose 1.54, $MgSO_4$ administration 1.67, and CCI-comorbidity 1.69.

The highest adjusted values were observed for intraoperative positioning (2.82), opioid induction (3.43), and intrathecal sufentanil (3.79). These findings suggest localized, non-trivial collinearity affecting a limited subset of perioperative strategy variables, without reaching thresholds typically considered problematic for model stability.

Model diagnostics were assessed using DHARMa simulated residuals. The scaled residuals did not deviate from the expected uniform distribution (Kolmogorov-Smirnov test: D = 0.048, p = 0.719). No evidence of over- or under-dispersion was detected (dispersion = 1.091, p = 0.462). No outliers were identified. Overall, DHARMa diagnostics did not indicate major violations of model assumptions.

2.1.1 Robust check analysis for total intraoperative opioid consumption rate and intrathecal regimen

The median intraoperative opioid consumption rate was 24.89 ng/kg/min (q_1-q_3 11.59-48.96, min-max 1.62-120.94).

Table S1 shows the results for univariate and multivariate gamma regression.

**Table S1**					
**Variable**		**Model intraoperative opioid consumption rate**			
		**Univariate**		**Multivariate model**	
		**β% (95% CI)**	**p-value**	**β% (95% CI)**	**p-value**
Age		-1.0 (-1.9 to -0.1)	0.032	-0.9 (-1.6 to -0.2)	0.010
Sex, male		-1.1 (-22. to +25.1)	0.926	+1.4 (-13.5 to +18.9)	0.860
BMI		-0.6 (-3.0 to +2.0)	0.642	-0.1 (-1.8 to +1.6)	0.867
Medication class burden	None	Ref.	0.399	Ref.	0.160
	One	-15.7 (-37.6 to +13.1)		+4.8 (-14.1 to +27.5)	
	Multiclass	-17.4 (-39.0 to +11.0)		-11.9 (-30.1 to +10.9)	
Smoking status	No	Ref.	<0.001	Ref.	<0.001
	Former	-1.6 (-25.4 to +31.1)		-8.2 (-22.8 to +9.5)	
	Current	+64.4 (+24.7 to +119.1)		+32.7 (+11.9 to +57.8)	
CCI-comorbidity		-3.0 (-9.1 to +3.8)	0.379	+5.6 (-1.3 to +13.2)	0.117
ASA class	I	Ref.	0.330	Ref.	0.489
	II	-20.7 (-56.1 to +31.5)		-18.7 (-45.4 to +19.0)	
	III	-31.1 (-62.6 to +17.3)		-15.0 (-46.9 to +34.7)	
Surgical site	Pelvic	Ref.	0.425	Ref.	0.519
	Retroperitoneal	+18.9 (-12.7 to +64.4)		+30.4 (-14.0 to +99.3)	
	Upper abdominal	+30.4 (-4.5 to +80.9)		+19.5 (-13.5 to +68.5)	
	Abdominal wall	+25.3 (-14.5 to +89.9)		+30.1 (-14.9 to +101.4)	
	Lower abdominal	+28.0 (-19.2 to +115.5)		+22.9 (-7.1 to +65.2)	
Intraoperative Trendelengurg, yes		-12.4 (-30.8 to +10.6)	0.265	+26.7 (-12.2 to +84.8)	0.208
Intrathecal Regimen	No-SA	Ref.	<0.001	Ref.	<0.001
	IM	+6.7 (-17.6-+37.7)		+17.0 (-8.3-+48.8)	
	IM+L5	-44.0 (-56.7- -27.7)		-52.8 (-63.3- -39.5)	
	IM+L5+S3	-56.2 (-66.5- -42.8)		-45.7 (-64.4- -19.4)	
	IM+L10+S3	-86.7 (-89.6- -83.0)		-80.5 (-87.0- -71.5)	
Opioid induction, Fentanyl		+185.9 (+129.0 to +256.3)	<0.001	+36.7 (-13.7 to +111.9)	0.179
Fentanyl-equivalent induction dose		+16.3 (-4.2 to +42.7)	0.130	+32.8 (+15.1 to +54.2)	<0.001
Propofol TCI, yes		+93.6 (+26.0 to +215.9)	0.002	+67.4 (+27.0 to +123.8)	<0.001
$MgSO_4$ administration, yes		-48.0 (-58.4 to -34.9)	<0.001	-5.3 (-24.3 to +18.1)	0.629
Clonidine administration, yes		+31.7 (-7.1 to +93.3)	0.125	+53.1 (+21.3 to +94.3)	<0.001
Intraoperative standardized blood loss		-4.0 (-16.8 to +16.8)	0.641	+8.1 (-3.8 to +24.0)	0.203

Multicollinearity was assessed using generalized variance inflation factors (VIF). Overall, no evidence of problematic multicollinearity was observed. Most adjusted VIF values were below 2, including age (1.39), sex (1.20), body mass index (1.20), medication class burden (1.24), smoking status (1.10), CCI-comorbidity (1.69), ASA physical status (1.37), surgical site (1.38), intrathecal regimen (1.47), fentanyl-equivalent induction dose (1.24), propofol TCI (1.11), $MgSO_4$ (1.67), clonidine administration (1.17), and standardized intraoperative blood loss (1.14). Slightly higher values were observed for intraoperative positioning (2.82) and induction opioid use (3.43), although both remained well below commonly accepted thresholds indicating problematic collinearity. Overall, these findings suggest that multicollinearity was unlikely to have materially affected the stability or interpretability of the regression model estimates.

Model diagnostics performed using the DHARMa package showed no evidence of model misspecification. The Kolmogorov-Smirnov test indicated that the scaled residuals did not significantly deviate from the expected uniform distribution ($D = 0.052$, $p = 0.623$), supporting an adequate overall model fit. The nonparametric dispersion test showed no evidence of overdispersion or underdispersion (dispersion = 0.846, $p = 0.474$). Likewise, the outlier test identified only one potential outlier among 207 observations, with a frequency not significantly different from that expected under the fitted model ($p = 0.339$). Overall, these diagnostic analyses did not reveal any major violations of the model assumptions, supporting the adequacy and robustness of the fitted regression model.

2.1.2 Provider-adjusted model

To account for potential clustering by treating anesthesiologist, a mixed-effects Gamma regression model with a random intercept for anesthesiologist was fitted. The estimated standard deviation of the random intercept was 0.268, indicating modest between-

anesthesiologist variability after adjustment for patient, surgical, and anesthetic characteristics.

Likelihood ratio tests showed that age ($p = 0.037$), smoking status ($p = 0.039$), intrathecal regimen ($p < 0.001$), fentanyl-equivalent induction dose ($p < 0.001$), propofol TCI ($p < 0.001$), and clonidine administration ($p = 0.001$) remained independently associated with AUC/h, whereas sex, BMI, CCI comorbidity, ASA physical status, surgical site, intraoperative positioning, induction opioid, $MgSO_4$, and intraoperative blood loss were not significant predictors.

Compared with the reference regimen (no intrathecal analgesia), IM alone was not associated with a significant reduction in opioid exposure (+4.8%, 95% CI -8.0% to +19.3%). In contrast, regimens containing levobupivacaine were associated with substantially lower opioid requirements, with reductions of 27.7% (95% CI -36.4% to -17.9%) for IM+L5, 28.3% (95% CI -41.1% to -12.7%) for IM+L5+S3, and 41.6% (95% CI -51.9% to -29.1%) for IM+L10+S3.

Current smoking was independently associated with a 10.2% increase in hourly opioid exposure (95% CI 1.9% to 19.3%). Each unit increase in fentanyl-equivalent induction dose was associated with a 54.2% increase in opioid exposure (95% CI 43.9% to 65.3%), while propofol TCI administration was associated with a 30.9% increase (95% CI 14.8% to 49.3%). Clonidine administration was independently associated with a 20.8% increase in opioid exposure (95% CI 8.0% to 35.0%). Overall, adjustment for anesthesiologist-level clustering did not materially change the magnitude, direction, or statistical significance of the main associations observed in the primary analysis, supporting the robustness of the findings.

2.2 AUC/h model and intrathecal drugs

Table S2 summarizes the univariable and multivariable Gamma regression analyses of factors associated with hourly intraoperative opioid exposure. In the adjusted model, increasing age was associated with a modest reduction in opioid requirements (-0.5% per

**Table S2**					
**Variable**		**Model AUC/h**			
		**Univariate**		**Multivariate model**	
		**β% (95% CI)**	**p-value**	**β% (95% CI)**	**p-value**
Age		-0.6 (-1.1 to -0.1)	0.014	-0.5 (-0.9 to -0.1)	0.021
Sex, male		-8.3 (-19.7 to +4.6)	0.197	-2.5 (-11.1 to +6.8)	0.583
BMI		-1.6 (-2.9 to -0.2)	0.022	-0.2 (-1.1 to +0.8)	0.712
Medication class burden	None	Ref.	0.280	Ref.	0.141
	One	-11.0 (-24.7 to +5.0)		+1.2 (-9.6 to +13.2)	
	Multiclass	-11.7 (-25.4 to +4.3)		-8.7 (-20.0 to +4.3)	
Smoking status	No	Ref.	<0.001	Ref.	0.002
	Former	-5.5 (-18.7 to +10.2)		-3.3 (-12.6 to +7.0)	
	Current	+35.3 (+16.4 to +57.7)		+17.2 (+6.2 to +29.5)	
CCI-comorbidity		-2.2 (-5.9 to +1.8)	0.277	+3.5 (-0.5 to +7.8)	0.087
ASA class	I	Ref.	0.160	Ref.	0.580
	II	-18.7 (-40.9 to +8.9)		-10.0 (-28.1 to +12.0)	
	III	-25.1 (-46.1 to +1.8)		-8.0 (-29.5 to +19.6)	
Surgical site	Pelvic	Ref.	0.272	Ref.	0.521
	Retroperitoneal	+18.8 (-0.1 to +42.1)		+14.5 (-9.8 to +45.7)	
	Upper abdominal	+15.6 (-3.0 to +38.5)		+4.5 (-13.3 to +26.7)	
	Abdominal wall	+9.0 (-12.2 to +36.8)		+12.7 (-11.7 to +44.4)	
	Lower abdominal	+13.9 (-12.5 to +51.0)		+11.3 (-5.5 to +31.9)	
Intraoperative Trendelenburg, yes		-8.6 (-19.8 to +4.1)	0.175	+13.0 (-8.4 to +39.7)	0.254
Intrathecal morphine, yes		-28.8 (-39.9 to -16.2)	<0.001	+14.0 (-0.9 to +31.1)	0.067
Intrathecal Levobupivacaine	No	Ref.	<0.001	Ref.	<0.001
	5.0 mg	-28.5 (-36.9 to -19.0)		-44.3 (-51.3 to -36.2)	
	10.0 mg	-50.3 (-56.8 to -42.7)		-59.3 (-66.0 to -51.3)	
Intrathecal sufentanil, yes		-35.1 (-42.2 to -27.0)	<0.001	+19.0 (-10.6 to +57.5)	0.231
Opioid induction, Fentanyl		+43.7 (+27.3 to +62.2)	<0.001	+6.0 (-18.3 to +36.5)	0.654
Fentanyl-equivalent induction dose		+39.3 (+25.1 to +55.5)	<0.001	+47.5 (+35.9 to +60.4)	<0.001
Propofol TCI, yes		+38.3 (+8.1 to +80.3)	0.009	+40.2 (+19.4 to +65.3)	<0.001
MgSO4 administration, yes		-16.0 (-26.1 to -4.5)	0.007	+0.1 (-11.8 to +13.4)	0.992
Clonidine administration, yes		+28.0 (+5.3 to +57.4)	0.013	+20.0 (+4.9 to +37.5)	0.008
Intraoperative standardized blood loss		+2.0 (-7.2 to +14.1)	0.704	+1.9 (-4.4 to +9.3)	0.576

year, 95% CI -0.9% to -0.1%; $p = 0.021$), whereas current smoking was independently associated with a 17.2% increase in opioid exposure (95% CI 6.2% to 29.5%; $p = 0.002$). Among the intrathecal analgesic components, levobupivacaine remained the only independent determinant of reduced opioid consumption, showing a clear dose-dependent association. Compared with no levobupivacaine, administration of 5 mg was associated with a 44.3% reduction in hourly opioid exposure (95% CI -51.3% to -36.2%), while 10 mg was associated with a 59.3% reduction (95% CI -66.0% to -51.3%; overall $p < 0.001$). By contrast, intrathecal morphine, intrathecal sufentanil, and fentanyl administered at induction were not independently associated with opioid exposure after adjustment.

Higher fentanyl-equivalent induction dose remained strongly associated with increased opioid exposure (+47.5%, 95% CI 35.9% to 60.4%; $p < 0.001$), as did the use of propofol TCI (+40.2%, 95% CI 19.4% to 65.3%; $p < 0.001$) and clonidine administration (+20.0%, 95% CI 4.9% to 37.5%; $p = 0.008$). Conversely, sex, BMI, medication class burden, CCI comorbidity, ASA physical status, surgical site, patient positioning, $MgSO_4$, and intraoperative blood loss were not independently associated with AUC/h.

2.2.1 VIF and DHARMA analyses

Multicollinearity was assessed using generalized VIF. Overall, no evidence of problematic multicollinearity was observed. Most adjusted VIF values were below 2, including age (1.39), sex (1.20), BMI (1.20), medication class burden (1.24), smoking status (1.10), CCI comorbidity (1.69), ASA physical status (1.37), surgical site (1.38), intrathecal morphine (1.44), levobupivacaine dose (1.54), fentanyl-equivalent induction dose (1.24), propofol-TCI (1.11), $MgSO_4$ (1.67), clonidine administration (1.17), and standardized intraoperative blood loss (1.14). Slightly higher values were observed for intraoperative positioning (2.82), induction opioid (3.43), and intrathecal sufentanil (3.79). However, all adjusted VIF values remained below the commonly accepted threshold of 5, indicating that multicollinearity was

unlikely to have materially affected coefficient estimation or the stability of the multivariable model.

Model diagnostics performed using the DHARMa package indicated an adequate model fit. The Kolmogorov-Smirnov test showed that the scaled residuals did not significantly deviate from the expected uniform distribution ($D = 0.045$, $p = 0.796$), supporting the assumption of correctly specified residuals. The nonparametric dispersion test found no evidence of overdispersion or underdispersion (dispersion = 1.229, $p = 0.172$). The outlier test identified only one potential outlier among 207 observations, with a frequency not significantly different from that expected under the fitted model ($p = 0.339$). Overall, the diagnostic analyses did not identify any substantial violations of model assumptions, supporting the adequacy of the fitted Gamma regression model.

2.2.2 Robust check analysis for total intraoperative opioid consumption rate and intrathecal drug administration

Table S3 shows the results for univariate and multivariate gamma regression, evaluating the association between intraoperative opioid consumption and intrathecal drug administration. The results were broadly consistent with those obtained for AUC/h. In the adjusted model, increasing age remained independently associated with a lower opioid consumption rate (-0.9% per year, 95% CI -1.6% to -0.2%; $p = 0.010$), whereas current smoking was associated with a 32.7% increase (95% CI 11.9% to 57.8%; $p < 0.001$).

Among the intrathecal analgesic components, levobupivacaine was again the only independent factor associated with reduced opioid requirements, demonstrating a marked dose-response relationship. Compared with no levobupivacaine, administration of 5 mg was associated with a 59.6% reduction in opioid consumption rate (95% CI -68.0% to -48.9%), while 10 mg was associated with an 85.5% reduction (95% CI -89.4% to -80.2%; overall p

< 0.001). In contrast, neither intrathecal morphine nor intrathecal sufentanil remained

**Table S3**					
**Variable**		**Model intraoperative opioid consumption rate**			
		**Univariate**		**Multivariate model**	
		**β% (95% CI)**	**p-value**	**β% (95% CI)**	**p-value**
Age		-1.0 (-1.9 to -0.1)	0.032	-0.9 (-1.6 to -0.2)	0.010
Sex, male		-1.1 (-22. to +25.1)	0.926	+1.4 (-13.5 to +18.9)	0.860
BMI		-0.6 (-3.0 to +2.0)	0.642	-0.1 (-1.8 to +1.6)	0.867
Medication class burden	None	Ref.	0.399	Ref.	0.160
	One	-15.7 (-37.6 to +13.1)		+4.8 (-14.1 to +27.5)	
	Multiclass	-17.4 (-39.0 to +11.0)		-11.9 (-30.1 to +10.9)	
Smoking status	No	Ref.	<0.001	Ref.	<0.001
	Former	-1.6 (-25.4 to +31.1)		-8.2 (-22.8 to +9.5)	
	Current	+64.4 (+24.7 to +119.1)		+32.7 (+11.9 to +57.8)	
CCI-comorbidity		-3.0 (-9.1 to +3.8)	0.379	+5.6 (-1.3 to +13.2)	0.117
ASA class	I	Ref.	0.330	Ref.	0.489
	II	-20.7 (-56.1 to +31.5)		-18.7 (-45.4 to +19.0)	
	III	-31.1 (-62.6 to +17.3)		-15.0 (-46.9 to +34.7)	
Surgical site	Pelvic	Ref.	0.425	Ref.	0.519
	Retroperitoneal	+18.9 (-12.7 to +64.4)		+30.4 (-14.0 to +99.3)	
	Upper abdominal	+30.4 (-4.5 to +80.9)		+19.5 (-13.5 to +68.5)	
	Abdominal wall	+25.3 (-14.5 to +89.9)		+30.1 (-14.9 to +101.4)	
	Lower abdominal	+28.0 (-19.2 to +115.5)		+22.9 (-7.1 to +65.2)	
Intraoperative Trendelenburg, yes		-12.4 (-30.8 to +10.6)	0.265	+26.7 (-12.2 to +84.8)	0.208
Intrathecal morphine, yes		-46.3 (-61.0 to -27.5)	<0.001	+17.0 (-8.3 to +48.8)	0.206
Intrathecal Levobupivacaine	No	Ref.	<0.001	Ref.	<0.001
	5.0 mg	-51.3 (-59.4 to -41.7)		-59.6 (-68.0 to -48.9)	
	10.0 mg	-87.2 (-89.5 to -84.2)		-85.5 (-89.4 to -80.2)	
Intrathecal sufentanil, yes		-70.0 (-75.6 to -62.9)	<0.001	+14.9 (-30.2 to +85.6)	0.579
Opioid induction, Fentanyl		+185.9 (+129.0 to +256.3)	<0.001	+36.7 (-13.7 to +111.9)	0.179
Fentanyl-equivalent induction dose		+16.3 (-4.2 to +42.7)	0.130	+32.8 (+15.1 to +54.2)	<0.001
Propofol TCI, yes		+93.6 (+26.0 to +215.9)	0.002	+67.4 (+27.0 to +123.8)	<0.001
$MgSO_4$ administration, yes		-48.0 (-58.4 to -34.9)	<0.001	-5.3 (-24.3 to +18.1)	0.629
Clonidine administration, yes		+31.7 (-7.1 to +93.3)	0.125	+53.1 (+21.3 to +94.3)	<0.001
Intraoperative standardized blood loss		-4.0 (-16.8 to +16.8)	0.641	+8.1 (-3.8 to +24.0)	0.203

independently associated with the outcome after multivariable adjustment.

Higher fentanyl-equivalent induction dose (+32.8%, 95% CI 15.1% to 54.2%; p < 0.001), target-controlled propofol infusion (+67.4%, 95% CI 27.0% to 123.8%; p < 0.001), and clonidine administration (+53.1%, 95% CI 21.3% to 94.3%; p < 0.001) were independently

associated with increased opioid consumption rate. No significant independent associations were observed for sex, BMI, medication class burden, CCI comorbidity, ASA physical status, surgical site, intraoperative positioning, $MgSO_4$, intraoperative blood loss, or induction opioid.

Adjusted multicollinearity diagnostics (VIF) were as follows: age 1.39, sex 1.20, BMI 1.20, medication class burden 1.24, smoking status 1.10, CCI-comorbidity 1.69, ASA-PS 1.37, surgical site 1.38, intraoperative positioning 2.82, intrathecal morphine 1.44, intrathecal levobupivacaine dose 1.54, intrathecal sufentanil 3.79, induction fentanyl 3.43, fentanyl-equivalent induction dose 1.24, propofol TCI 1.11, $MgSO_4$ administration 1.67, clonidine administration 1.17, and intraoperative standardized blood loss 1.14.

Model diagnostics were assessed using DHARMa simulated residuals. The scaled residuals did not deviate from the expected uniform distribution (Kolmogorov-Smirnov test: D = 0.052, p = 0.623). No evidence of over- or under-dispersion was detected (dispersion = 0.846, p = 0.474). DHARMa diagnostics did not indicate an excess of outliers (exact binomial outlier test: 1 outlier out of 207 observations; observed frequency 0.48% vs expected 0.20%; p = 0.339). Overall, DHARMa diagnostics did not indicate major violations of model assumptions.

2.3 Intraoperative standardized fluid administration and intrathecal regimen model

No evidence of severe multicollinearity was observed when interpreting the adjusted VIFs (all VIFs < 5). Overall, diagnostics indicated limited multicollinearity among most covariates. Several predictors exhibited adjusted values close to 1, consistent with negligible shared variance (e.g., smoking 1.10, TCI 1.11, intraoperative standardized blood loss 1.14, clonidine 1.17, BMI 1.20, sex 1.20, medication class burden 1.24, and fentanyl-equivalent dose 1.24). Most multi-level factor variables also showed low adjusted values, including ASA physical status (1.37) and surgical site (1.38).

A smaller subset of predictors showed moderate adjusted values, indicating some shared variance with other model terms but remaining within acceptable ranges for multivariable clinical regression. These included intrathecal morphine 1.44, intrathecal levobupivacaine dose 1.54, $MgSO_4$ administration 1.67, and CCI-comorbidity 1.69. Age also showed a mildly elevated adjusted value (1.39), still well below common concern thresholds.

The highest adjusted VIF values were observed for intraoperative positioning (2.82), opioid induction (3.43), and intrathecal sufentanil (3.79). These results suggest localized, non-trivial collinearity affecting a limited subset of perioperative strategy variables, without reaching thresholds typically considered problematic for model stability.

DHARMa diagnostics based on simulated scaled residuals did not indicate major violations of model assumptions. Residuals were consistent with the expected uniform distribution (Kolmogorov-Smirnov test: $D = 0.0570$, $p = 0.512$), and no evidence of over- or under-dispersion was detected (dispersion = 0.985, $p = 0.926$). The outlier test identified 1 observation out of 207 (observed frequency 0.00483 vs expected 0.001998), with no significant excess of outliers ($p = 0.339$).

2.3.1 Provider-adjusted model

To account for potential clustering by treating anesthesiologist, a mixed-effects Gamma regression model with a random intercept for anesthesiologist was fitted. The estimated standard deviation of the random intercept was 0.080, indicating minimal between-anesthesiologist variability after adjustment for patient, surgical, and anesthetic characteristics.

Likelihood ratio tests showed that BMI ($p < 0.001$), CCI comorbidity ($p < 0.001$), intrathecal regimen ($p = 0.002$), and standardized intraoperative blood loss ($p < 0.001$) remained independently associated with standardized intraoperative fluid administration. Age, sex, medication class burden, smoking status, intraoperative positioning, induction opioid,

fentanyl-equivalent induction dose, propofol TCI, $MgSO_4$, clonidine administration, and ASA physical status were not independently associated with fluid requirements after adjustment. Compared with the reference group (no SA), the IM regimen alone was not associated with a significant reduction in fluid administration (-11.7%, 95% CI -25.3% to +4.3%). In contrast, the regimen combining IM+L5 was associated with a 22.1% reduction in standardized fluid administration (95% CI -34.0% to -8.0%), while the regimen IM+L10+S3 was associated with a 30.8% reduction (95% CI -46.4% to -10.7%). The regimen IM+L5+S3 showed a similar direction of effect (-18.8%, 95% CI -37.3 to +5.2%) but did not reach statistical significance. Higher BMI was independently associated with lower fluid administration (-3.1% per kg/m^2; 95% CI, -4.2% to -2.0%), whereas increasing CCi comorbidity was associated with a 7.2% reduction (95% CI, -11.1% to -3.1%) in standardized fluid requirements. Conversely, greater standardized intraoperative blood loss was associated with a 17.9% increase in fluid administration (95% CI 9.1% to 27.5%). Overall, adjustment for anesthesiologist-level clustering did not materially alter the magnitude, direction, or statistical significance of the principal associations observed in the primary analysis, supporting the robustness of the findings.

2.4 Intraoperative standardized fluid administration and intrathecal drug administration

Table S4 shows the results for univariate and multivariate gamma regression, evaluating the association between intraoperative standardized fluid administration and intrathecal drug administration.

In the adjusted model, higher BMI was independently associated with lower fluid administration (-2.9% per kg/m^2, 95% CI -4.0% to -1.8%; $p < 0.001$), while increasing CCi comorbidity was associated with a 7.0% reduction (95% CI -11.1% to -2.6%; $p = 0.002$). As

expected, greater standardized intraoperative blood loss remained independently associated with increased fluid administration (+18.1%, 95% CI 9.2% to 28.9%; p < 0.001).

Table S4

**Variable**		**Model standardized intraoperative fluid administration**			
		**Univariate**		**Multivariate model**	
		**β% (95% CI)**	**p-value**	**β% (95% CI)**	**p-value**
Age		-0.4 (-0.8 to 0.0)	0.048	+0.1 (-0.4 to +0.5)	0.782
Sex, male		-16.3 (-25.1 to -6.5)	0.002	-3.0 (-12.7 to +7.7)	0.567
BMI		-3.5 (-4.5 to -2.4)	<0.001	-2.9 (-4.0 to -1.8)	<0.001
Medication class burden	None	Ref.	0.172	Ref.	0.582
	One	-6.4 (-18.8 to +7.7)		+4.6 (-8.0 to +18.7)	
	Multiclass	-12.6 (-24.3 to +0.7)		-1.2 (-15.3 to +15.1)	
Smoking status	No	Ref.	0.792	Ref.	0.784
	Former	-1.0 (-13.7 to +13.9)		+3.3 (-7.8 to +15.9)	
	Current	+4.3 (-9.1 to +19.9)		+3.2 (-7.9 to +15.8)	
CCI-comorbidity		-5.4 (-8.4 to -2.3)	<0.001	-7.0 (-11.1 to -2.6)	0.002
ASA class	I	Ref.	0.010	Ref.	0.120
	II	-27.3 (-43.8 to -7.6)		+12.5 (-12.9 to + 44.1)	
	III	-31.7 (-47.7 to -12.2)		+29.0 (-4.7 to +73.8)	
Surgical site	Pelvic	Ref.	0.016	Ref.	0.148
	Retroperitoneal	+12.8 (-2.8 to +31.3)		+14.2 (-13.2 to +50.9)	
	Upper abdominal	+29.7 (+11.6 to +51.2)		+24.6 (+0.5 to +55.8)	
	Abdominal wall	+6.5 (-11.5 to +29.2)		+9.9 (-16.9 to +46.1)	
	Lower abdominal	+9.3 (-12.8 to +38.9)		+13.6 (-6.0 to +38.1)	
Intraoperative Trendelenburg, yes		-10.9 (-20.3 to -0.4)	0.043	+12.1 (-12.0 to +43.4)	0.357
Intrathecal morphine, yes		-15.9 (-27.6 to -2.9)	0.018	-8.5 (-22.1 to +7.3)	0.276
Intrathecal Levobupivacaine	No	Ref.	0.290	Ref.	0.001
	5.0 mg	-2.7 (-14.3 to +10.4)		-17.9 (-29.6 to -4.1)	
	10.0 mg	-10.8 (-22.7 to +3.1)		-31.5 (-44.1 to -16.0)	
Intrathecal sufentanil, yes		+9.9 (-1.6 to +22.9)	0.094	+10.1 (-20.5 to +51.2)	0.559
Opioid induction, Fentanyl		-13.1 (-22.0 to -3.2)	0.011	-17.4 (-38.4 to +9.6)	0.187
Fentanyl-equivalent induction dose		+12.4 (+1.8 to +24.5)	0.021	+0.2 (-8.6 to +10.2)	0.969
Propofol TCI, yes		-3.5 (-22.1 to +21.3)	0.755	-4.3 (-20.3 to +15.7)	0.647
$MgSO_4$ administration, yes		+5.1 (-5.9 to +17.4)	0.378	+1.9 (-11.6 to +17.2)	0.798
Clonidine administration, yes		+2.4 (-13.6 to +22.3)	0.787	-2.6 (-16.5 to +13.8)	0.735
Intraoperative standardized blood loss		+24.8 (+14.4 to +37.8)	<0.001	+18.1 (+9.2 to +28.9)	<0.001

Among the intrathecal analgesic components, only levobupivacaine remained independently associated with reduced fluid requirements. Compared with no levobupivacaine, administration of 5 mg was associated with a 17.9% reduction in standardized fluid administration (95% CI -29.6% to -4.1%), whereas 10 mg was associated with a 31.5% reduction (95% CI -44.1% to -16.0%; overall p = 0.001), demonstrating a dose-dependent association. In contrast, intrathecal morphine and intrathecal sufentanil were not independently associated with fluid administration after adjustment for potential confounders.

No independent associations were observed for age, sex, medication class burden, smoking status, ASA physical status, surgical site, intraoperative positioning, induction opioid, fentanyl-equivalent induction dose, target-controlled propofol infusion, magnesium sulfate administration, or clonidine administration.

Multicollinearity was assessed using generalized variance inflation factors (VIF). No evidence of problematic multicollinearity was identified. Most adjusted VIF values were below 2, including age (1.39), sex (1.20), BMI (1.20), medication class burden (1.24), smoking status (1.10), CCI comorbidity (1.69), ASA physical status (1.37), surgical site (1.38), intrathecal morphine (1.44), levobupivacaine dose (1.54), fentanyl-equivalent induction dose (1.24), propofol TCI (1.11), $MgSO_4$ (1.67), clonidine administration (1.17), and standardized intraoperative blood loss (1.14). Slightly higher adjusted VIF values were observed for intraoperative positioning (2.82), induction opioid (3.43), and intrathecal sufentanil (3.79), although all remained below the commonly accepted threshold of 5. Overall, these findings indicate that multicollinearity was unlikely to have materially influenced coefficient estimation or the stability of the multivariable model.

Model diagnostics performed using the DHARMa package demonstrated an adequate fit of the multivariable Gamma regression model. The Kolmogorov-Smirnov test showed no significant deviation of the scaled residuals from the expected uniform distribution ($D = 0.057$, $p = 0.512$), indicating no evidence of model misspecification. The nonparametric dispersion test found no evidence of either overdispersion or underdispersion (dispersion = 0.985, $p = 0.926$). The outlier test identified only one potential outlier among 207 observations, and its frequency did not differ significantly from that expected under the fitted model ($p = 0.339$). Overall, the diagnostic analyses did not reveal any substantial violations of model assumptions, supporting the adequacy and robustness of the fitted model.

2.5 Comparison of patients with complete vs incomplete NRS data

To assess the potential impact of missing postoperative pain assessments, patients with complete NRS measurements at all predefined time points were compared with those with incomplete NRS data. Continuous variables were compared using the Mann-Whitney test, while categorical variables were compared using Pearson's chi-square test or Fisher's exact test, as appropriate according to expected cell frequencies.

Table S5 compares patients with complete vs. incomplete NRS data.

**Table S5**				
**Variable**		**Complete NRS (n=159)**	**Incomplete NRS (n=48)**	**p-value**
Age (years)		66.0 (55.0-71.0)	65.0 (54.7-71.0)	0.737
Sex, male (%)		100 (62.9)	29 (60.4)	0.888
BMI (kg/m^2)		26.3 (24.1-29.7)	26.6 (24.6-30.7)	0.372
Comorbidities				
Solid Tumor	No (%)	46 (28.9)	16 (33.3)	0.152
	Localized (%)	112 (70.4)	30 (62.5)	
	Metastatic (%)	1 (0.6)	2 (4.2)	
Hypertension (%)		98 (61.6)	32 (66.7)	0.644
Smoking	No (%)	86 (54.1)	25 (52.1)	0.943
	Former (%)	36 (22.6)	12 (25.0)	
	Current (%)	37 (23.3)	11 (22.9)	
Obesity (%)		38 (23.9)	14 (29.2)	0.584
COPD (%)		32 (20.1)	7 (14.6)	0.516
Diabetes	No (%)	141 (88.7)	40 (83.3)	0.494
	Uncomplicated (%)	17 (10.7)	7 (14.6)	
	End-organ injury (%)	1 (0.6)	1 (2.1)	
Myocardial infarction (%)		8 (5.0)	5 (10.4)	0.436
Atrial fibrillation (%)[F]		6 (3.8)	3 (6.2)	0.739
Autoimmune disease (%)[F]		6 (3.8)	2 (4.2)	1.000
CKD (%)[F]		2 (1.2)	2 (4.2)	0.230
PVD (%)[F]		4 (2.5)	0 (0.0)	0.575
CVA (%)[F]		4 (2.5)	0 (0.0)	0.575
Mild cognitive impairment (%)[F]		3 (1.9)	1 (2.1)	1.000
Mild liver disease (%)[F]		0 (0.0)	1 (2.1)	0.232
Leukemia/Lymphoma (%)[F]		0 (0.0)	1 (2.1)	0.232
HIV infection (%)[F]		1 (0.6)	0 (0.0)	1.000
Home-medications				
Cardiovascular (%)		107 (67.3)	33 (68.7)	0.990
Antiaggregant/anticoagulant (%)		39 (24.5)	13 (27.1)	0.867

Antidiabetic (%)		20 (12.6)	8 (16.7)	0.628
Psychotropic (%)[F]		16 (10.1)	3 (6.2)	0.573
Thyroid (%)[F]		11 (6.9)	4 (8.3)	0.753
Medication class burden	None (%)	38 (23.9)	12 (25.0)	0.868
	One (%)	63 (39.6)	17 (35.4)	
	Multiclass (%)	58 (36.5)	19 (39.6)	
CCI score (points)		4.0 (2.0-5.5)	4.0 (2.7-5.0)	0.905
CCI-comorbidity (points)		2.0 (0.5-3.0)	2.0 (1.7-3.0)	0.865
ASA class	I (%)	9 (5.7)	1 (2.1)	0.591
	II (%)	104 (65.4)	32 (66.7)	
	III (%)	46 (28.9)	15 (31.2)	
Surgical and intraoperative management				
Operated organ	Prostate (%)	61 (38.4)	10 (20.8)	0.004
	Kidney (%)	29 (18.2)	6 (12.5)	
	Uterus (%)	23 (14.4)	2 (4.2)	
	Colon-rectus (%)	13 (8.2)	9 (18.7)	
	Abdominal wall (%)	13 (8.2)	8 (16.7)	
	Gallbladder (%)	10 (6.3)	8 (16.7)	
	Other (%)	10 (6.3)	5 (10.4)	
Surgical site	Pelvic (%)	86 (54.1)	12 (25.0)	<0.001
	Retroperitoneal (%)	31 (19.5)	7 (14.6)	
	Upper abdominal (%)	20 (12.6)	17 (35.4)	
	Abdominal wall (%)	13 (8.2)	8 (16.7)	
	Lower abdominal (%)	9 (5.7)	4 (8.3)	
Intraoperative Trendelenburg (%)		98 (61.6)	21 (43.7)	0.042
Surgical time (min)		210.0 (162.5-250.0)	210.0 (167.5-243.8)	0.879
Anesthesia time (min)		240.0 (195.0-280.0)	273.5 (188.8-276.2)	0.799
Subarachnoid analgesia (%)		135 (84.9)	37 (77.1)	0.295
Intrathecal morphine (%)		135 (84.9)	37 (77.1)	0.295
Intrathecal Levobupivacaine	No	45 (28.3)	33 (68.7)	<0.001
	5.0 mg	70 (44.0)	9 (18.7)	
	10.0 mg	44 (27.7)	6 (12.5)	
Intrathecal sufentanil (%)		75 (47.2)	11 (22.9)	0.005
Opioid induction	Fentanyl (%)	77 (48.4)	37 (77.1)	<0.001
	Sufentanil (%)	82 (51.6)	11 (22.9)	
Fentanyl equivalent dose (mcg/kg)		1.5 (1.2-1.9)	1.3 (1.1-1.5)	0.010
Propofol TCI (%)		7 (4.4)	7 (14.6)	0.033
$MgSO_4$ administration (%)		101 (63.5)	10 (20.8)	<0.001
Clonidine administration (%)		24 (15.1)	0 (0.0)	0.009
Intraoperative remifentanil infusion (%)		114 (71.7)	38 (79.2)	0.401
Model-derived intraoperative opioid AUC (ng·h/mL)		24.6 (18.6-33.1)	35.0 (22.0-44.5)	0.001

Postoperative elastomeric pump	33 (20.7)	18 (37.5)	0.030
Intraoperative fluid management			
Colloid administration (%)^{F}	16 (10.1)	0 (0.0)	0.026
PRBC administration (%)^{F}	2 (1.2)	0 (0.0)	1.000
Standardized fluid administration (mL/kg/h)	5.37 (3.89-6.60)	5.47 (4.44-6.57)	0.344
Standardized urine output (mL/kg/h)	0.71 (0.47-1.03)	0.81 (0.62-1.58)	0.017
Standardized blood loss (mL/kg/h)	0.58 (0.42-0.79)	0.58 (0.39-0.74)	0.566
Standardized fluid output (mL/kg/h)	1.75 (1.42-2.18)	1.85 (1.59-2.49)	0.119
Standardized fluid balance (mL/kg/h)	3.38 (2.25-4.29)	3.38 (2.41-4.47)	0.712

Overall, the two groups showed broadly comparable demographic characteristics, comorbidity burden, baseline clinical status, and intraoperative fluid management variables. However, significant differences emerged in the surgical setting and perioperative analgesic strategies. Patients with complete NRS data more frequently underwent pelvic surgery and Trendelenburg positioning and were more likely to receive intrathecal levobupivacaine, intrathecal sufentanil, $MgSO_4$, and clonidine. Conversely, patients with incomplete NRS data showed higher model-derived intraoperative opioid exposure and more frequent use of postoperative elastomeric analgesia.

Given the exploratory and tertiary nature of postoperative pain-related analyses in the present study, no additional formal sensitivity analyses for missing outcome data were performed.

2.6 Free-pain model

Adjusted VIFs were uniformly low across predictors, supporting limited collinearity within the multivariable model. The lowest adjusted VIFs were observed for smoking (1.05), surgical site (1.07), and BMI (1.08), followed by neuroaxial analgesia (1.13), postoperative analgesic mode (1.14), and sex (1.15). Medication class burden and ASA physical status showed slightly higher, though still modest, adjusted values (1.24 and 1.27, respectively). Age remained within the low-to-moderate range (1.39). The highest adjusted VIF was observed for CCI-comorbidity (1.65), while all remaining predictors demonstrated similarly acceptable

values. Overall, these adjusted VIFs do not indicate meaningful multicollinearity and support the numerical stability and interpretability of the multivariable estimates.

The DHARMa diagnostics did not identify major violations of model assumptions. The uniformity test indicated that the simulated scaled residuals were consistent with the expected uniform distribution (Kolmogorov-Smirnov D = 0.056, p = 0.692), supporting an adequate overall fit. Likewise, the dispersion test did not suggest over- or under-dispersion (dispersion = 1.010, p = 0.862), which is reassuring for the binomial-logit specification. Finally, the outlier test detected no influential outliers.

2.6.1 Provider random effect analysis for free-pain

In the provider-adjusted mixed-effects logistic regression model, the overall pattern of associations remained consistent with the primary multivariable analysis. In likelihood-ratio single-term deletion (drop-one) testing, only CCI-comorbidity (p = 0.034) and SA (p = 0.040) retained independent associations with the pain-free outcome after accounting for provider-level clustering, whereas the remaining covariates were not significant (age p = 0.255, sex p = 0.933, BMI p = 0.346, medication class burden p = 0.324, smoking status p = 0.937, ASA-PS p = 0.983, surgical site p = 0.499, postoperative analgesia mode p = 0.640). Consistent with the main analysis, CCI-comorbidity remained associated with higher odds of pain-free status (OR 1.52, 95% CI 1.02-2.27), while SA showed the strongest association with the endpoint (OR 3.33, 95% CI 1.01-10.95). These findings support the robustness of the associations for CCI-comorbidity and SA beyond between-anesthesiologist variability.

Reference

1. Olson MT, Lombardi L, Clarke W. Clinical consequences of analytical variance and calculation strategy in oral busulfan pharmacokinetics. *Clin Chim Acta*. Nov 20 2011;412(23-24):2316–21. doi:10.1016/j.cca.2011.08.028

2. Byers JP, Sarver JG. Pharmacokinetic Modeling. In: Hacker M, Messer W, Bachmann K, eds. *Pharmacology*. Academic Press; 2009:201–277.

3. Palmer PP, Miller RD. Current and developing methods of patient-controlled analgesia. *Anesthesiology Clinics*. 2010;28(4):587–599.
